# The interferon landscape along the respiratory tract impacts the severity of COVID-19

**DOI:** 10.1016/j.cell.2021.08.016

**Published:** 2021-09-16

**Authors:** Benedetta Sposito, Achille Broggi, Laura Pandolfi, Stefania Crotta, Nicola Clementi, Roberto Ferrarese, Sofia Sisti, Elena Criscuolo, Roberto Spreafico, Jaclyn M. Long, Alessandro Ambrosi, Enju Liu, Vanessa Frangipane, Laura Saracino, Sara Bozzini, Laura Marongiu, Fabio A. Facchini, Andrea Bottazzi, Tommaso Fossali, Riccardo Colombo, Massimo Clementi, Elena Tagliabue, Janet Chou, Antonio E. Pontiroli, Federica Meloni, Andreas Wack, Nicasio Mancini, Ivan Zanoni

**Affiliations:** 1 Harvard Medical School, Boston Children’s Hospital, Division of Immunology, Boston, MA 02115, USA; 2 Department of Biotechnology and Biosciences and Ph.D. Program in Molecular and Translational Medicine (DIMET), University of Milano - Bicocca, Milan 20100, Italy; 3 Respiratory Disease Unit IRCCS San Matteo Hospital Foundation, Pavia 27100, Italy; 4 Immunoregulation Laboratory, The Francis Crick Institute, London NW1 1AT, UK; 5 Laboratory of Medical Microbiology and Virology, Vita-Salute San Raffaele University, Milan 20100, Italy; 6 IRCCS San Raffaele Hospital, Milan 20100, Italy; 7 Institute for Quantitative and Computational Biosciences, University of California, Los Angeles, Los Angeles, CA, USA; 8 Faculty of Medicine and Surgery, Vita-Salute San Raffaele University, Milan 20100, Italy; 9 Harvard Medical School, Boston Children’s Hospital, Division of Gastroenterology, Boston, MA 02115, USA; 10 Institutional Centers for Clinical and Translational Research, Boston Children’s Hospital, Boston, MA 02115, USA; 11 Department of Anesthesia and Critical Care Medicine, IRCCS Policlinico San Matteo Foundation, Pavia 27100, Italy; 12 Division of Anesthesiology and Intensive Care, ASST Fatebenefratelli Sacco, Luigi Sacco Hospital, University of Milan, Milan 20100, Italy; 13 Value-based healthcare unit, IRCCS Multimedica, Milan 20100, Italy; 14 Department of Health Sciences, University of Milan, Milan 20100, Italy; 15 Department of Internal Medicine and Pharmacology, University of Pavia, Pavia 27100, Italy

**Keywords:** interferon, Type I IFN, Type III IFN, COVID-19, SARS-CoV-2, dendritic cell, epithelial cell, pattern recognition receptor, lung, airways

## Abstract

Severe coronavirus disease 2019 (COVID-19) is characterized by overproduction of immune mediators, but the role of interferons (IFNs) of the type I (IFN-I) or type III (IFN-III) families remains debated. We scrutinized the production of IFNs along the respiratory tract of COVID-19 patients and found that high levels of IFN-III, and to a lesser extent IFN-I, characterize the upper airways of patients with high viral burden but reduced disease risk or severity. Production of specific IFN-III, but not IFN-I, members denotes patients with a mild pathology and efficiently drives the transcription of genes that protect against severe acute respiratory syndrome coronavirus 2 (SARS-CoV-2). In contrast, compared to subjects with other infectious or noninfectious lung pathologies, IFNs are overrepresented in the lower airways of patients with severe COVID-19 that exhibit gene pathways associated with increased apoptosis and decreased proliferation. Our data demonstrate a dynamic production of IFNs in SARS-CoV-2-infected patients and show IFNs play opposing roles at distinct anatomical sites.

## Highlights

• IFN expression in COVID-19 varies based on location, viral load, age, and severity
• IFN-λ1 and IFN-λ3 drive protective ISGs in the upper airways of mildly ill patients
• Critical patients express IFN-αβ and IFN-λ2 and have low ISGs and high p53 expression
• Epithelial cells efficiently produce protective IFN-λ1, while cDCs express IFN-λ2,3

## Graphical abstract

**Figure:**
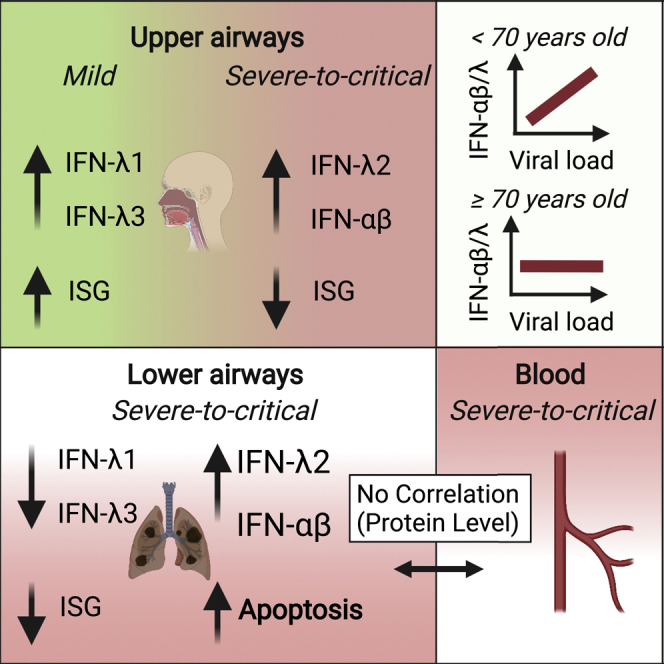

## Teaser

An in-depth analysis of IFNs in COVID-19 reveals differences in their roles based on anatomical location, viral load, age, and disease severity. In the upper respiratory tract, high levels of IFN-III are protective and result in mild disease in spite of higher SARS-CoV-2 viral burden, while the lower airways of patients with severe COVID-19 demonstrate elevated IFN-I and IFN-III, cell death, and a reduction in IFN-stimulated genes.

## Introduction

Since the outbreak of the coronavirus disease 2019 (COVID-19) in late 2019, the novel, severe acute respiratory syndrome coronavirus 2 (SARS-CoV-2) has infected over 188 million people globally and caused more than 4 million deaths as of July 2021. SARS-CoV-2 infection can lead to acute respiratory distress syndrome (ARDS) characterized by elevated levels of proinflammatory cytokines in the bloodstream (Guan et al., 2020; Lee et al., 2020; Lucas et al., 2020; Zhou et al., 2020a). Mouse models and retrospective human studies suggest that severity and death following a SARS-CoV-2 encounter is correlated with exaggerated inflammation rather than viral load (Bergamaschi et al., 2021; Guan et al., 2020; Karki et al., 2021; Lee et al., 2020; Lucas et al., 2020; Ruan et al., 2020; Winkler et al., 2020; Zhou et al., 2020a). Nevertheless, how a balance between the benefits (restricting viral replication and spread) and risks (inducing a cytokine storm) of efficient immune cell activation is achieved during COVID-19 remains a mystery.

Of the many inflammatory mediators produced upon infection with SARS-CoV-2, interferons (IFNs) have attracted much attention since the inception of the pandemic. IFNs belong to three major families: type I (IFN-I; mainly represented by IFN-αs and IFN-β), IFN-II (IFN-γ), and IFN-III (IFN-λ1-4). Upregulation of IFN-II in patients with severe COVID-19 (Karki et al., 2021; Lucas et al., 2020) is associated with increased PANoptosis, which exacerbates pathology and death (Karki et al., 2021). In contrast, the roles of IFN-I and IFN-III during SARS-CoV-2 infection have been a matter of debate. Indeed, IFN-I and IFN-III exert potent antiviral functions via the induction of IFN-stimulated genes (ISGs). Several studies showed that SARS-CoV-2, compared to other viruses, boosts the production of inflammatory mediators while delaying and/or dampening antiviral IFN responses in patients with severe COVID-19 (Blanco-Melo et al., 2020; Galani et al., 2021; Hadjadj et al., 2020; Mudd et al., 2020). Nevertheless, regulation of IFN-I and IFN-III production following infection with SARS-CoV-2 appears to be more complex. In fact, analyses of nasopharyngeal swabs (Cheemarla et al., 2021; Lieberman et al., 2020; Ziegler et al., 2021), bronchoalveolar lavage fluid (BALF) (Zhou et al., 2020b), or peripheral blood monocytes (Lee et al., 2020) of COVID-19 patients have revealed potent ISG induction. Production of IFNs is also sustained in the blood of a longitudinal cohort of severe COVID-19 patients compared to subjects with a mild illness (Lucas et al., 2020).

Aside from the challenge of understanding the pattern of expression of IFNs, a major unanswered question is whether IFNs serve protective or detrimental functions in COVID-19. Recent studies show that patients with severe COVID-19 have defective IFN responses (Bastard et al., 2020; Combes et al., 2021; Laing et al., 2020; Pairo-Castineira et al., 2021; Wang et al., 2021; Zhang et al., 2020). Other studies, however, report that heightened and prolonged production of IFNs in patients infected with SARS-CoV-2 is correlated with negative clinical outcomes (Lee et al., 2020; Lucas et al., 2020). We and others have also recently demonstrated that the production of IFN-III, and to a lesser extent IFN-I, impairs lung function and may trigger a severe disease in mouse models of lung viral infections (Broggi et al., 2020a; Major et al., 2020). Thus, it is urgent to fully unravel the role of IFNs in the pathogenesis of COVID-19.

To define how IFN production impacts the progression of COVID-19, here, we analyzed the pattern and level of expression of IFNs and the transcriptional programs associated with the IFN landscape in the upper or lower respiratory tract of COVID-19 patients, subjects with infectious and noninfectious lung diseases, and healthy controls.

## Results

### High viral loads drive the efficient production of IFN-III, and to a lesser extent IFN-I, in an age-dependent manner in the upper airways of COVID-19 patients

**Figure S1:**
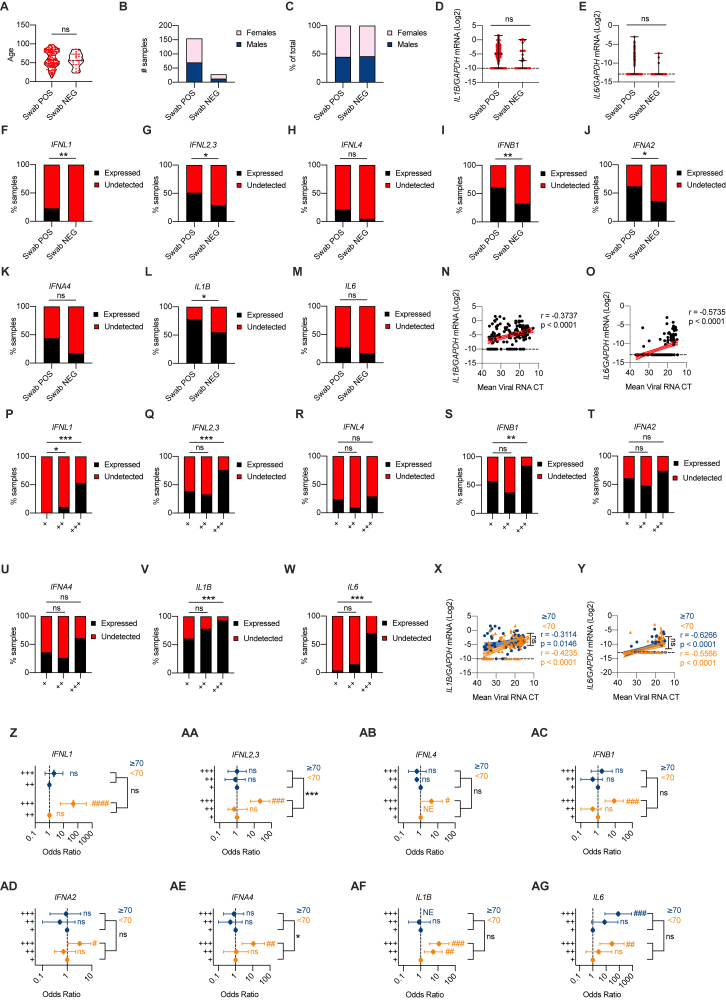
High viral loads drive the efficient production of IFN-III and, to a lesser extent, IFN-I in an age-dependent manner in the upper airways of COVID-19 patients, related to Figure 1 (A-C) Age distribution (A), number (B) and percentage (C) of females and males in cohorts of patients (Swab NEG, Swab POS) analyzed in Figures 1A–1L and Figures S1D–S1O. (A) Each dot represents a patient. Violin plots are depicted. (D, E) *IL1B* (D), and *IL6* (E) mRNA expression was evaluated in nasopharyngeal swabs from SARS-CoV-2-negative (Swab NEG) and -positive (Swab POS) subjects. Each dot represents a patient. Median with range is depicted. Dashed line represents limit of detection. (F-M) Percentage of patients that express (Expressed, black bars) or not (Undetected, red bars) *IFNL1* (F*)*, *IFNL2*,*3* (G*)*, *IFNL4* (H), *IFNB1* (I), *IFNA2* (J), *IFNA4* (K), *IL1B* (L), and *IL6* (M) in Swab POS and Swab NEG cohorts. (N-O) *IL1B* (N), and *IL6* (O) mRNA expression is plotted against mean viral RNA CT in Swab POS cohorts. Each dot represents a patient. Linear regression lines (continuous line) and 95% confidence interval (dashed line and shaded area) are depicted in red. Spearman correlation coefficients (r) and p value (p) are indicated. (P-W) Percentage of patients that express (Expressed, black bars) or not (Undetected, red bars) *IFNL1* (P*)*, *IFNL2*,*3* (Q*)*, *IFNL4* (R), *IFNB1* (S), *IFNA2* (T), *IFNA4* (U), *IL1B* (V), and *IL6* (W) in viral load tercile cohorts (“+,” “++,” “+++”). (X, Y) *IL1B* (X), and *IL6* (Y) mRNA expression is plotted against mean viral RNA CT in swabs from SARS-CoV-2 positive patients over 70-year-old (≥70, blue dots and lines) and below 70-year-old (< 70, orange dots and lines). Each dot represents a patient. Linear regression (continuous lines), 95% confidence interval (dashed line and shaded area), Spearman correlation coefficients (r) and p value (p) are indicated in blue and in orange for ≥ 70 and < 70 year-old patients respectively. (Z-AG) Odds ratio of expressing *IFNL1* (Z) mRNA in “+++” with respect to “++” SARS-CoV-2 positive swabs and *IFNL2*,*3* (AA*)*, *IFNL4* (AB), *IFNB1* (AC), *IFNA2* (AD), *IFNA4* (AE), *IL1B* (AF), and *IL6* (AG) mRNA in “+++” and “++” with respect to “+” SARS-CoV-2 positive swabs in ≥ 70 (blue dots and lines) and < 70 (orange dots and lines) patients. Symbols represent the odds ratio. Error bars represent the 95% confidence interval associated to the odds ratio. NE: not estimable, AB) no patient in group expresses *IFNL4*, AF) all patients in group express *IL1B*. (D, E, N, O, X, Y) Expression is plotted as log2 (*gene*/*GAPDH* mRNA + 0.5 x gene-specific minimum). Statistics: (A) Unpaired t test: ns, not significant (p > 0.05); ^∗^p < 0.05, ^∗∗^p < 0.01, ^∗∗∗^p < 0.001, and ^∗∗∗∗^p < 0.0001. (D, E) Mann-Whitney test: ns, not significant (p > 0.05); ^∗^p < 0.05, ^∗∗^p < 0.01, ^∗∗∗^p < 0.001, and ^∗∗∗∗^p < 0.0001. (F-M and P-W) Fisher’s exact test with Bonferroni correction: ns, not significant (p > 0.05); ^∗^p < 0.05, ^∗∗^p < 0.01, ^∗∗∗^p < 0.001, and ^∗∗∗∗^p < 0.0001. (Z-AG) Odds ratio: ns, not significant (p > 0.05); #p < 0.05, ##p < 0.01, ###p < 0.001. Interaction analysis: ns, not significant (p > 0.05); ^∗^p < 0.05, ^∗∗^p < 0.01, ^∗∗∗^p < 0.001. See also Tables S1 and S2.

**Figure 1:**
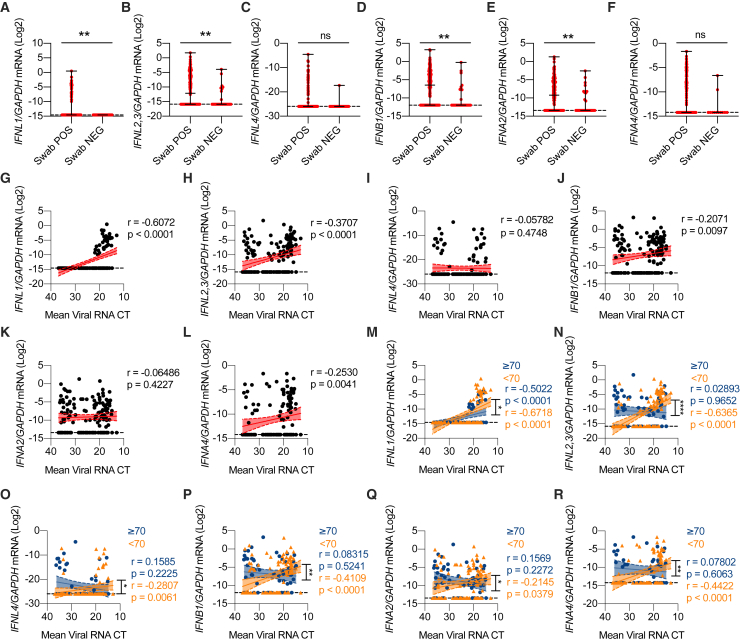
High viral loads drive the efficient production of IFN-III and, to a lesser extent, IFN-I in an age-dependent manner in the upper airways of COVID-19 patients (A–F) *IFNL1* (A), *IFNL2*,*3* (B), *IFNL4* (C), *IFNB1* (D), *IFNA2* (E), and *IFNA4* (F) mRNA expression was evaluated in nasopharyngeal swabs from SARS-CoV-2-negative (Swab NEG; 28) and positive (Swab POS; 155) subjects. Each dot represents a patient. Median with range is depicted. Dashed line represents limit of detection. (G–L) *IFNL1* (G), *IFNL2*,*3* (H), *IFNL4* (I), *IFNB1* (J), *IFNA2* (K), and *IFNA4* (L) mRNA expression is plotted against mean viral RNA cycle threshold (CT) in swabs from SARS-CoV-2-positive patients (155). Each dot represents a patient. Linear regression lines (continuous line) and 95% confidence interval (dashed line and shaded area) are depicted in red. Spearman correlation coefficients (r) and p value (p) are indicated. Dashed horizontal black line represents limit of detection. (M–R) *IFNL1* (M), *IFNL2,3* (N), *IFNL4* (O), *IFNB1* (P), *IFNA2* (Q), and *IFNA4* (R) mRNA expression is plotted against mean viral RNA CT in swabs from SARS-CoV-2-positive patients aged ≥70 years (61, blue dots and lines) and <70 years (94, orange dots and lines). Each dot represents a patient. Linear regression (continuous lines) and 95% confidence interval (dashed line and shaded area) are depicted. Spearman correlation coefficients (r) and p value (p) are indicated in blue and orange for patients ≥70 and <70 years, respectively. Dashed horizontal black line represents limit of detection. (A–R) Expression is plotted as log2 (*gene*/*GAPDH* mRNA + 0.5 × gene-specific minimum). Statistics by Mann-Whitney test: ns, not significant (p > 0.05); ^∗^p < 0.05; ^∗∗^p < 0.01; ^∗∗∗^p < 0.001; ^∗∗∗∗^p < 0.0001 (A–F) or test for difference between simple linear regression slopes: ns, not significant (p > 0.05); ^∗^p < 0.05; ^∗∗^p < 0.01; ^∗∗∗^p < 0.001; ^∗∗∗∗^p < 0.0001 (M–R). See also Figure S1 and Table S1.

We initially analyzed IFN gene expression in nasopharyngeal swabs derived from SARS-CoV-2-positive and negative subjects (Table S1; Figures S1A–S1C) and found that in subjects positive for SARS-CoV-2, *IFNL1* and *IFNL2,3* (among IFN-IIIs) and *IFNB1* and *IFNA2* (among IFN-Is) were significantly upregulated (Figures 1A–1F). As controls, *IL1B* and *IL6* were also analyzed in the same cohort of subjects (Figures S1D and S1E). To account for the bimodal distribution of cytokine gene expression, we transformed gene expression data in discrete variables (expressed or undetected) and obtained results similar to what we observed with continuous gene expression (Figures S1F–S1M).

Next, we examined the distribution of IFN levels relative to the viral load. Of the IFN-III family members, IFN-λ1 and IFN-λ2,3 positively correlated with viral load (Figures 1G–1I). Among IFN-Is, IFN-β and IFN-α4 also showed a positive correlation with the viral load (Figures 1J–1L). Transcript levels of the proinflammatory cytokines *IL1B* and *IL6* were also positively correlated with the viral load (Figures S1N–S1O). Next, we divided our patient cohort into terciles based on the viral load (Table S1) and analyzed gene expression using discrete variables. These analyses confirmed that IFN-λ1, IFN-λ2,3, IFN-β, interleukin-1β (IL-1β), and IL-6 were preferentially expressed in high (compared to low) viral load samples (Figures S1P–S1W).

We then evaluated how IFN gene expression relates to the age of patients, a key determinant of severity and lethality of COVID-19 (McPadden et al., 2020; Williamson et al., 2020). Our analyses demonstrated that IFN-III and IFN-I expression is significantly associated with viral load for the cohort of patients aged <70 years (Figures 1M–1R). In contrast, IFN expression in the cohort of patients aged ≥70 years either completely lost association with the viral load and/or showed a significantly lower correlation coefficient compared to the <70 years cohort (Figures 1M–1R). IL-1β and IL-6 maintained their association with viral load independent of age and were not significantly different in the two age cohorts (Figures S1X and S1Y). When we analyzed gene expression as a discrete variable, we found that response patterns to viral load were significantly different between elderly (≥70 years) and younger (<70 years) patients for IFN-λ2,3 and IFN-α4 (Figures S1Z–S1AE; Table S2). This analysis also showed that only younger patients have a dose-response relationship between IFN gene expression and viral load. In contrast to IFNs, no difference in the dose-response relationship between IL-1β and IL-6 expression and viral load was observed between age groups (Figures S1AF and S1AG; Table S2). These results indicate that in COVID-19 patients, the production of IFNs correlates with the viral load in the upper respiratory tract and that elderly patients, who are at risk of developing severe disease, have dysregulated IFN induction, which correlates more loosely with the viral load, compared to younger patients.

### Mild COVID-19 is characterized by high levels of IFN-III, but not IFN-I, in response to high viral loads in the upper airways

**Figure 2:**
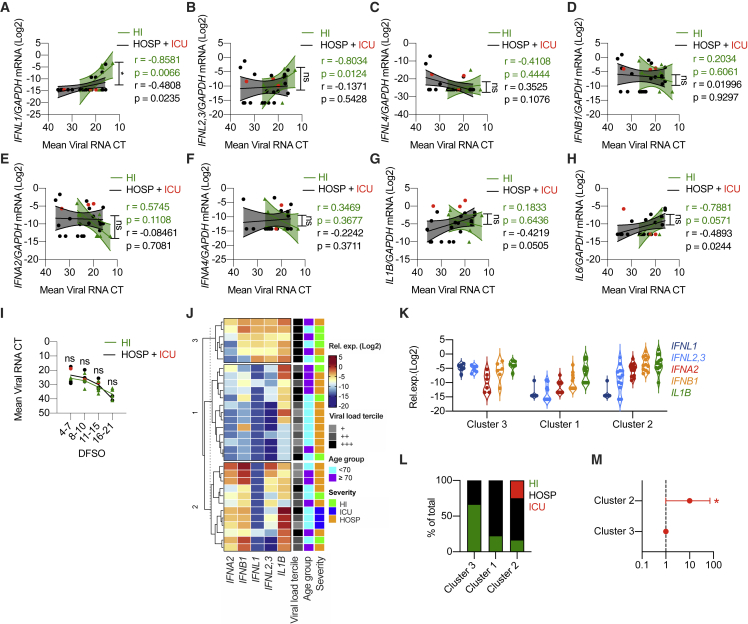
Mild COVID-19 is characterized by high levels of IFN-III, but not IFN-I, in response to high viral loads in the upper airways (A–M) Swabs from a cohort of SARS-CoV-2-positive hospitalized patients and ICU inpatients (HOSP, black dots; ICU, red dots; both HOSP and ICU, black lines and analyzed together) and home-isolated patients (HI, green dots and lines) were analyzed. (A–H) *IFNL1* (A), *IFNL2,3* (B), *IFNL4* (C), *IFNB1* (D), *IFNA2* (E), *IFNA4* (F), *IL1B* (G), and *IL6* (H) mRNA expression is plotted against mean viral RNA CT. Each dot represents a patient. Linear regression lines (continuous line) and 95% confidence interval (dashed line and shaded area) are depicted. Spearman correlation coefficients (r) and p value (p) are indicated in black and in green for “HOSP + ICU” and “HI” patients respectively. (I) Mean viral RNA CT values are plotted against days from symptom onset (DFSO). Each dot represents a patient. Lines connect mean values for each range of DFSO. (J) K-means clustering based on the expression of *IFNA2*, *IFNB1 IFNL1*, *IFNL2*,*3*, and *IL1B* was used to determine clusters 1–3 (cluster 1, n = 13; cluster 2, n = 12; cluster 3, n = 6). The color indicates the relative gene expression. Viral load tercile, age group, and severity are annotated. Viral load terciles (“+++,” “++,” and “+”) are defined by mean viral RNA CT (<20, >20 and <30, and >30). Age groups are defined as <70 or ≥70 years. (K) *IFNL1*, *IFNL2*,*3*, *IFNA2*, *IFNB1*, and *IL1B* mRNA expression within clusters identified in (J). Each dot represents a patient. Violin plots are depicted. (L) Percentage of patients with the indicated disease severity within clusters identified in (J). (M) Odds ratio of patients in cluster 2 being hospitalized or admitted to the ICU relative to patients in cluster 3 (clusters identified in J). Symbols represent the odds ratio. Error bars represent the 95% confidence interval associated with the odds ratio. (A–H and K) Expression is plotted as log2 (*gene*/*GAPDH* mRNA *+* 0.5 × gene-specific minimum). Statistics by test for difference between simple linear regression slopes: ns, not significant (p > 0.05); ^∗^p < 0.05; ^∗∗^p < 0.01; ^∗∗∗^p < 0.001; ^∗∗∗∗^p < 0.0001 (A–H); two-way ANOVA: ns, not significant (p > 0.05); ^∗^p < 0.05; ^∗∗^p < 0.01; ^∗∗∗^p < 0.001; ^∗∗∗∗^p < 0.0001 (I); or chi-square test for odds ratio: ns, not significant (p > 0.05); ^∗^p < 0.05; ^∗∗^p < 0.01; ^∗∗∗^p < 0.001 (L). See also Figure S2 and Table S3.

To explore the link between IFN production and disease severity, we analyzed nasopharyngeal swabs from a subset of patients with known clinical follow-up. Disease severity was assessed as follows: patients with mild disease manifestations discharged from the emergency room without being hospitalized (home isolated [HI]), severe patients who required hospitalization (hospitalized [HOSP]), and critically ill patients admitted to the intensive care unit (ICU) (Table S3). When gene expression levels were plotted against the viral load in HI versus HOSP/ICU (Figures 2A–2H), patients with a mild disease showed a positive correlation with expression of several members of the IFN-III family (Figures 2A–2C). In HOSP/ICU patients, this correlation was lost for IFN-λ2,3 and was significantly reduced for IFN-λ1 compared to HI patients (Figures 2A and 2B). In contrast to IFN-III, the positive correlation between *IL6* levels and viral load was maintained only for HOSP and ICU patients (Figure 2H). When members of the IFN-I family or *IL1B* expression was analyzed, no positive correlation was found in either hospitalized or HI patients (Figures 2D–2G). To control for possible differences due to random sampling, we assessed how the viral load varies based on the day from symptom onset in patients with different disease severity (Table S3) and found no significant difference (Figure 2I).

**Figure S2:**
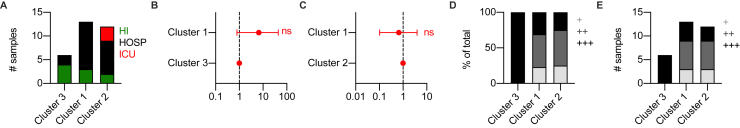
Mild COVID-19 is characterized by high levels of IFN-III, but not IFN-I, in response to high viral loads in the upper airways, related to Figure 2 (A) Number of samples from each disease severity group (HI = home-isolated, HOSP = hospitalized and ICU = Intensive care unit) within each cluster identified in Figure 2J. (B-C) Odds ratio of patients in Cluster 1 being hospitalized or admitted to the ICU relative to patients in Cluster 3 (B) and Cluster 2 (C) (Clusters identified in Figure 2J). Symbols represent the odds ratio. Error bars represent the 95% confidence interval associated to the odds ratio. (D-E) Percentage (D) and number (E) of samples from each viral load tercile (“+++,” “++,” “+”) within each cluster identified in Figure 2J. Viral load terciles (“+++,” “++,” “+”) are defined by mean viral RNA CT (< 20, > 20 and < 30, > 30). Statistics: (B-C) Chi Square test for odds ratio: ns, not significant (p > 0.05); ^∗^p < 0.05, ^∗∗^p < 0.01, ^∗∗∗^p < 0.001. See also Table S3.

To further investigate the distribution of IFN-III production in subjects with mild, severe, or critical illness, we performed K-mean clustering based on the expression of IFN-I, IFN-III, and IL-1β. Our results reveal that cluster 3, characterized by the highest expression of IFN-III, was enriched in patients with milder disease manifestations and high viral load (Figures 2J–2M and S2A–S2E). Notably, patients in cluster 2 (characterized by low levels of IFN-III and the highest levels of IFN-I) were 10 times more likely to have severe illness resulting in hospitalization or ICU admission than patients in cluster 3, and patients in cluster 1 (that presented low IFN-I and IFN-III expression and high IL-1β expression) showed a similar trend (Figures 2J–2M and S2A–S2C). Overall, these data support the hypothesis that efficient production of IFN-III in the upper airways of COVID-19 patients with high viral load protects against severe COVID-19.

### IFN-λ1 and IFN-λ3, but not IFN-λ2 or IFN-I, characterize the upper airways of patients with mild COVID-19 and drive ISGs that protect against SARS-CoV-2

**Figure 3:**
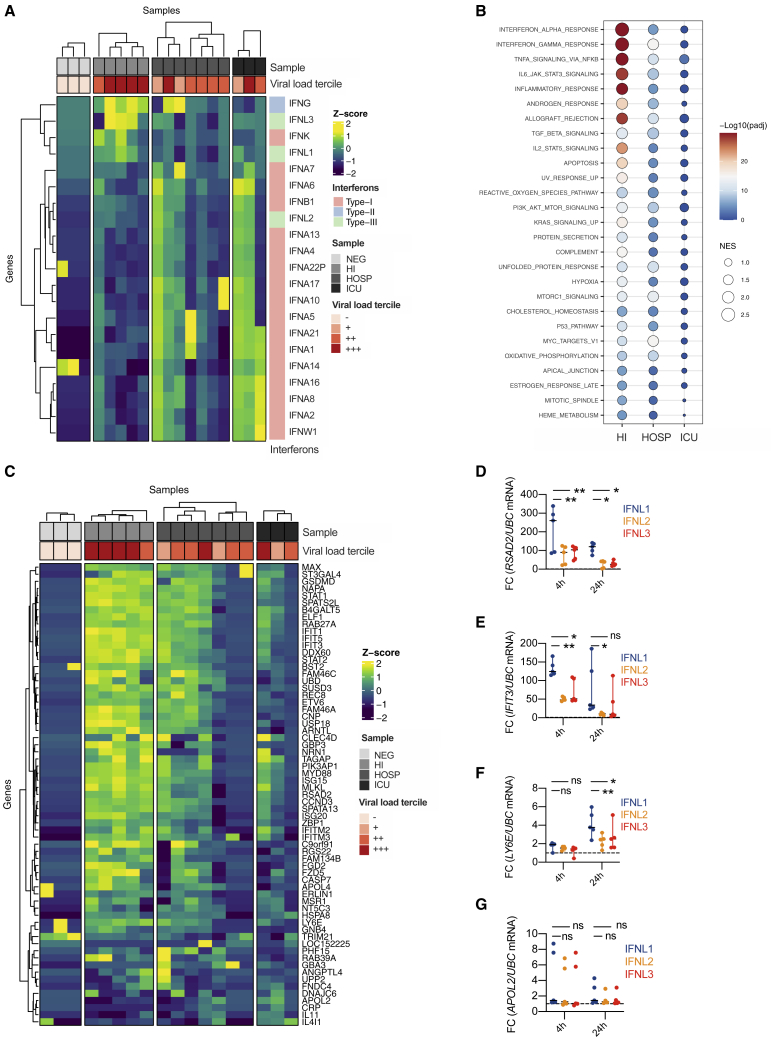
IFN-λ1 and IFN-λ3, but not IFN-λ2 or IFN-I, characterize the upper airways of patients with mild COVID-19 and drive ISGs that protect against SARS-CoV-2 (A–C) Targeted RNA-seq of nasopharyngeal swabs from SARS-CoV-2-negative (NEG; 3) and positive patients with known disease severity: home-isolated patients (HI; 5), hospitalized patients (HOSP; 7), ICU inpatients (ICU; 3). (A) Heatmap depicting expression of IFN-I/IFN-II/IFN-III. The color is proportional to the *Z* score. (B) Bubble plot visualization of gene set enrichment analysis (GSEA) for pathways enriched in HI, HOSP, and ICU patients. Normalized enrichment score (NES) is depicted. Color coding corresponds to −log10 (p adjusted value [padj]). Pathways with padj < 0.05 in either group are represented. (C) Heatmap depicting expression of ISGs that protect against SARS-CoV-2. The color is proportional to the *Z* score. (D–G) Human bronchial epithelial cells (hBECs) were treated with human recombinant IFN-λ1, IFN-λ2, or IFN-λ3 at a concentration of 2 ng/mL for 4 or 24 h. *RSAD2* (D) *IFIT3* (E), *LY6E* (F), and *APOL2* (G) mRNA expression was evaluated. Each dot represents a biological replicate. Median with range is depicted. FC, fold change compared to untreated cells. Statistics by two-way ANOVA: ns, not significant (p > 0.05); ^∗^p < 0.05; ^∗∗^p < 0.01; ^∗∗∗^p < 0.001; ^∗∗∗∗^p < 0.0001. See also Figure S3 and Table S4.

**Figure S3:**
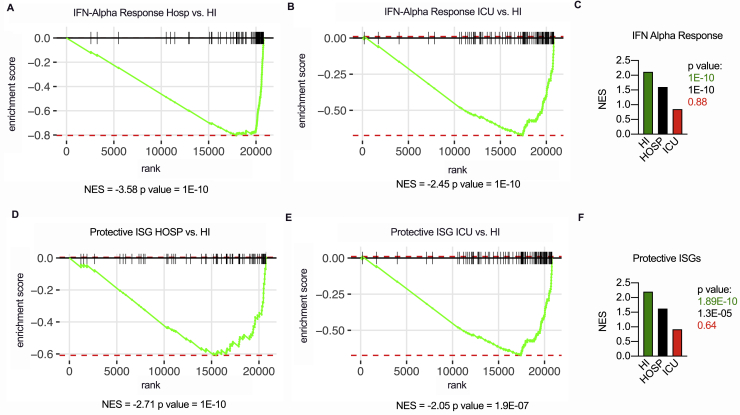
IFN-λ1 and IFN-λ3, but not IFN-λ2 or IFN-I, characterize the upper airways of patients with mild COVID-19 and drive ISGs that protect against SARS-CoV-2, related to Figure 3 (A-F) Targeted RNA-sequencing of nasopharyngeal swabs from SARS-CoV-2 negative (NEG, 3) and positive patients with known disease severity: home-isolated patients (HI, 5), hospitalized patients (HOSP, 7), ICU inpatients (ICU, 3). (A-B) Gene set enrichment analysis (GSEA) enrichment plot for genes belonging to the interferon alpha response (HALLMARK Pathways) between HOSP and HI (A) and ICU and HI (B) cohorts of patients. (C) Normalized enrichment score (NES) and p value of interferon alpha response geneset (HALLMARK Pathways) in HI, HOSP and ICU patients as compared to NEG. (D-E) GSEA enrichment plot for protective ISG geneset (Curated Geneset derived from Martin-Sancho et al., 2021) between HOSP and HI (D) and ICU and HI (E) cohorts of patients. (F) Normalized enrichment score (NES) of protective ISG geneset in HI, HOSP and ICU patients as compared to NEG. See also Table S4.

To gain more insight into the transcriptional programs linked to expression of specific IFN members, we used targeted RNA sequencing (RNA-seq) to examine the swabs of a subset of COVID-19 patients (Table S4). We found that IFN-λ1 and IFN-λ3 (now distinguishable from IFN-λ2 because of sequencing) segregated with subjects with mild COVID-19 and a high viral load compared to healthy controls or more severely ill COVID-19 patients (Figure 3A). IFN-γ was expressed in patients with mild and severe COVID-19, while IFN-I and IFN-λ2 were mostly associated with critical, and to a lesser extent severe, patients (Figure 3A). When gene set enrichment analysis was performed, the IFN responses were the most significantly enriched in subjects with mild (compared to severe or critical) COVID-19 (Figures 3B, S3A, and S3B). When compared to swabs from SARS-CoV-2 negative subjects, patients with mild and severe, but not critical, COVID-19 were enriched in IFN responses (Figure S3C). To determine whether the pattern of IFNs found in HI patients drove a protective response against SARS-CoV-2, we tested expression of >50 ISGs that directly restrain SARS-CoV-2 infection (Martin-Sancho et al., 2021). RNA-seq data demonstrate that only patients with mild manifestations efficiently upregulated this set of protective ISGs (Figures 3C, S3D, and S3E) and that this set of ISGs was significantly enriched compared to controls (Figure S3F).

Due to the high sequence identity of the IFN-III family members (Broggi et al., 2020b), we next compared the capacity of IFN-λ1, IFN-λ2, and IFN-λ3 to induce specific ISGs. We stimulated human bronchial epithelial cells (hBECs) with different IFN-IIIs and found that IFN-λ1 induces and sustains the transcription of several ISGs more efficiently than IFN-λ2 and, to some extent, IFN-λ3 (Figures 3D–3G). Overall, our data demonstrate that specific members of the IFN families associate with mild or severe COVID-19, that the landscape of IFNs determines the ISGs induced in the upper airways, and that IFN-λ1 is uniquely capable of inducing potent anti-SARS-CoV-2 ISGs in patients with mild COVID-19.

### Members of the IFN-III and IFN-I families are overrepresented in the lower airways of COVID-19 patients

**Figure S4:**
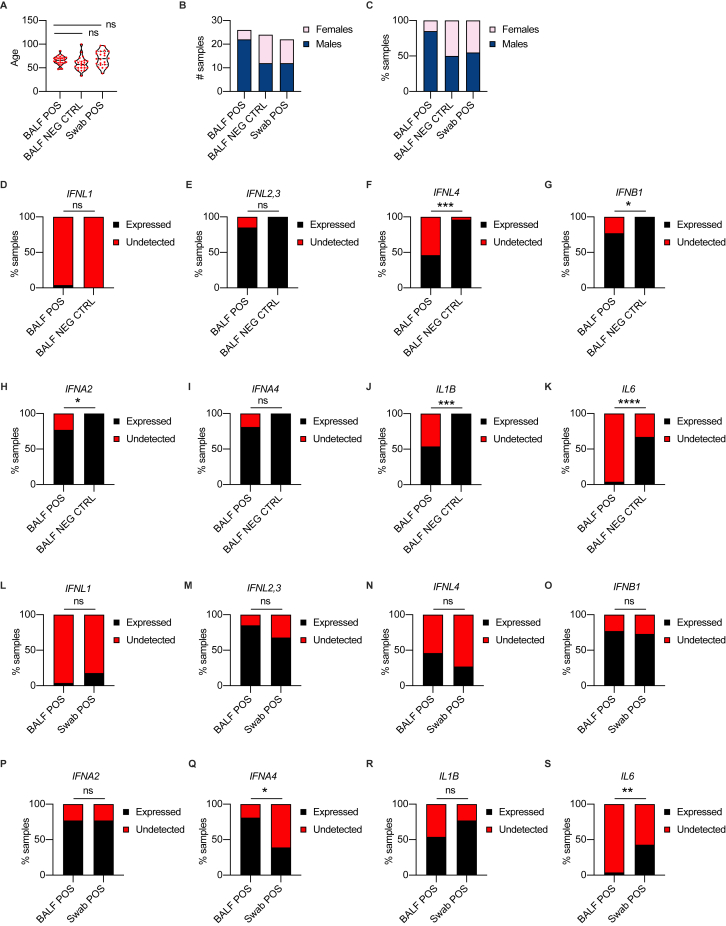
Members of the IFN-III and IFN-I families are overrepresented in the lower airways of COVID-19 patients, related to Figure 4 (A-C) Age distribution (A), number (B) and percentage (C) of females and males in cohorts of patients (BALF POS, BALF NEG CTRL and Swab POS) analyzed in Figure 4A-P. (A) Each dot represents a patient. Violin plots are depicted. (D-K) Percentage of patients in BALF from SARS-CoV-2-positive (BALF POS, 26) and -negative (BALF NEG CTRL, 24) that express (Expressed, black bars) or not (Undetected, red bars) *IFNL1* (D*)*, *IFNL2*,*3* (E*)*, *IFNL4* (F), *IFNB1* (G), *IFNA2* (H), *IFNA4* (I), *IL1B* (J), and *IL6* (K). (L-S) Percentage of patients (BALF POS, 26) and swabs (Swab POS, 21) from SARS-CoV-2-positive subjects that express (Expressed, black bars) or not (Undetected, red bars) *IFNL1* (L*)*, *IFNL2*,*3* (M*)*, *IFNL4* (N), *IFNB1* (O), *IFNA2* (P), *IFNA4* (Q), *IL1B* (R), and *IL6* (S). Statistics: (D-S) Fisher’s exact test: ns, not significant (p > 0.05); ^∗^p < 0.05, ^∗∗^p < 0.01, ^∗∗∗^p < 0.001, and ^∗∗∗∗^p < 0.0001. See also Table S5.

A detailed analysis of the IFNs produced in the lower airways of SARS-CoV-2-infected subjects is lacking. We thus analyzed BALF samples derived from COVID-19 HOSP patients, including ICU-admitted subjects, and, as controls, samples derived from patients with noninfectious lung pathologies (see Table S5 and Figures S4A–S4C for details regarding sex and age distribution).

**Figure 4:**
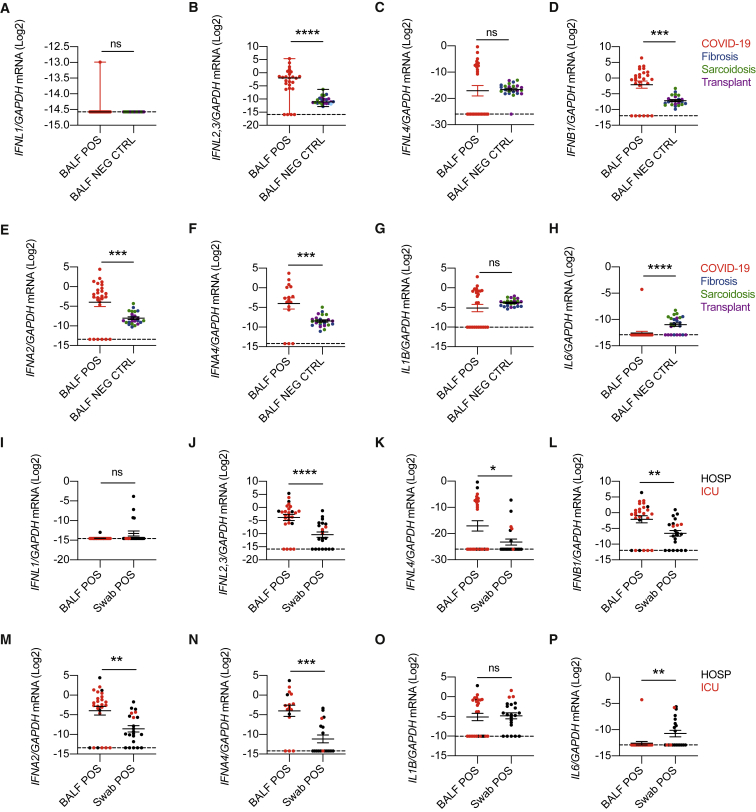
Members of the IFN-III and IFN-I families are overrepresented in the lower airways of COVID-19 patients (A–H) *IFNL1* (A), *IFNL2,3* (B), *IFNL4* (C), *IFNB1* (D), *IFNA2* (E), *IFNA4* (F), *IL1B* (G), and *IL6* (H) mRNA expression was evaluated in BALF from SARS-CoV-2-positive (BALF POS; 26, red dot) and negative (BALF NEG CTRL; 24) patients with noninfectious lung involvement such as fibrosis (8, blue dot), sarcoidosis (8, green dot), or lung transplant (8, purple dot). (I–P) *IFNL1* (I), *IFNL2,3* (J), *IFNL4* (K), *IFNB1* (L), *IFNA2* (M), *IFNA4* (N), *IL1B* (O), and *IL6* (P) mRNA expression was evaluated in BALF (BALF POS; 26) and swabs (Swab POS; 21) from SARS-CoV-2-positive subjects who were either hospitalized (HOSP; black dots) or ICU inpatients (ICU; red dots). (A–P) Expression is plotted as log2 (*gene*/*GAPDH* mRNA + 0.5 × gene-specific minimum). Each dot represents a patient. Median with range is depicted. Statistics by Mann-Whitney test: ns, not significant (p > 0.05); ^∗^p < 0.05; ^∗∗^p < 0.01; ^∗∗∗^p < 0.001; ^∗∗∗∗^p < 0.0001. See also Figure S4 and Table S5.

Transcripts of IFN-λ2,3, IFN-β, IFN-α2, and IFN-α4 were significantly upregulated in COVID-19 patients compared to controls (Figures 4A–4F), while a similar percentage of subjects expressed the genes analyzed (Figures S4D–S4I). No difference was observed for *IL1B* transcripts, while *IL6* mRNA levels appeared to be slightly increased in controls compared to COVID-19 patients (Figures 4G, 4H, S4J, and S4K).

We next compared the expression of IFNs between the lower and upper airways of COVID-19 patients with similar disease severity. Sex and age were distributed as reported in Table S5. We found that, except for IFN-λ1, levels of IFNs in severe-to-critical patients were higher in the lower compared to the upper airways (Figures 4I–4N), while a similar percentage of patients expressed IFNs in the upper or lower respiratory tract, except for IFN-α4 (Figures S4L–S4Q). *IL1B* mRNA levels were not different in the upper and lower airways of hospitalized COVID-19 patients, while *IL6* transcripts appeared to be predominantly expressed in the nasopharyngeal swabs compared to the BALF (Figures 4O, 4P, S4R, and S4S). These data demonstrate that selected members of both IFN-III and IFN-I families are overrepresented in the lower airways compared to the upper airways of hospitalized COVID-19 patients.

### Critical COVID-19 is characterized by the induction of a similar IFN landscape in the upper and lower airways

**Figure 5:**
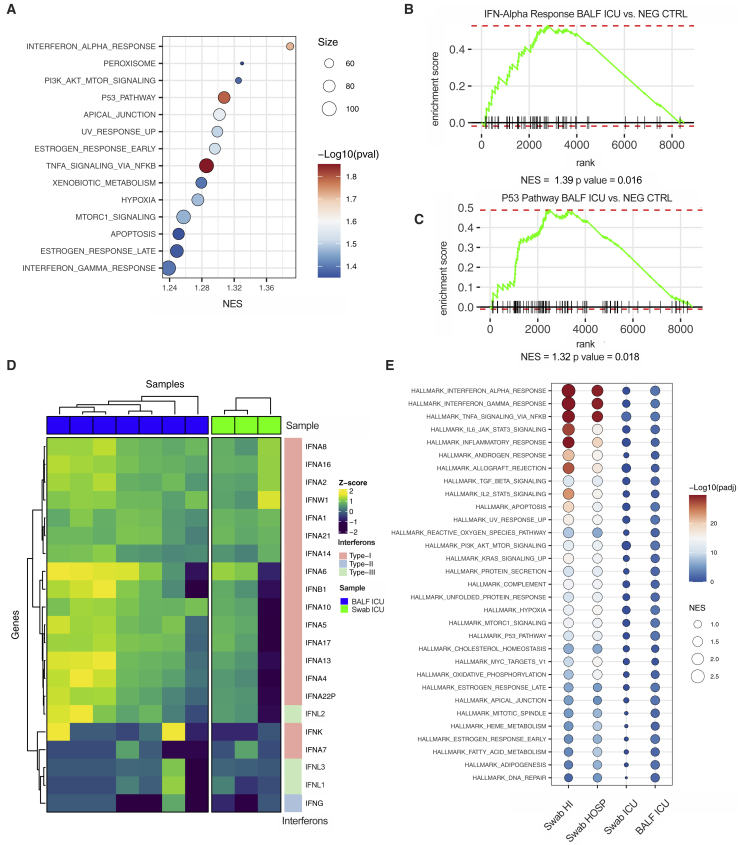
Critical COVID-19 is characterized by the induction of a similar IFN landscape in the upper and lower airways (A–E) Targeted RNA-seq of BALF from SARS-CoV-2-positive patients (BALF ICU; 7), patients with noninfectious lung pathologies (BALF NEG CTRL; 5), and nasopharyngeal swabs from SARS-CoV-2-positive patients who were either ICU inpatients (Swab ICU; 3), hospitalized (Swab HOSP; 7), or HI (Swab HI; 5). The color is proportional to the *Z* score. (A) Bubble plot visualization of GSEA for pathways enriched in BALF ICU compared to BALF NEG CTRL samples. NES is depicted. Color coding corresponds to −log10(p adjusted value [padj]), and size corresponds to the number of genes detected for each pathway. Pathways with p value (pval)< 0.05 are depicted. (B and C) GSEA enrichment plot for genes belonging to the interferon alpha response (B) and p53 pathway (C) in BALF ICU and BALF NEG CTRL samples. (D) Heatmap depicting expression of IFN-I/IFN-II/IFN-III IFNs in BALF ICU and Swab ICU samples. (E) Dot plot visualization of GSEA for pathways enriched in the lower airways of critical patients (BALF ICU) and the upper airways of patients with different disease severity (Swab HI, Swab HOSP, and Swab ICU). NES is depicted. Color coding corresponds to −log10(padj). Pathways with padj < 0.05 in any of the groups are depicted. See also Figure S5 and Table S6.

**Figure S5:**
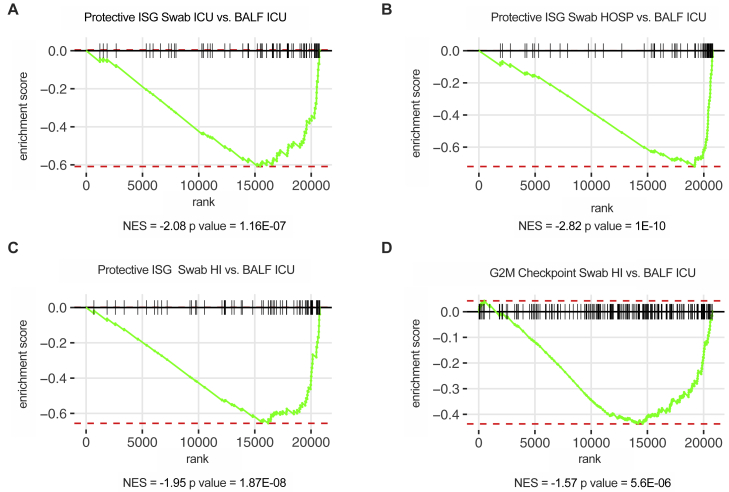
Critical COVID-19 is characterized by the induction of a similar IFN landscape in the upper and lower airways, related to Figure 5 (A-C) Targeted RNA-sequencing of BALF from SARS-CoV-2 positive patients (BALF ICU, 7), and from nasopharyngeal swabs from SARS-CoV-2 positive patients that were either ICU inpatients (Swab ICU, 3) hospitalized (Swab HOSP, 7) or home-isolated (Swab HI, 5). (A-C) GSEA enrichment plot for protective ISG genes (curated Geneset derived from Martin-Sancho et al., 2021) between Swab ICU and BALF ICU (A), Swab HOSP and BALF ICU (B), Swab HI and BALF ICU (C). (D) GSEA enrichment plot for genes involved in the G2M checkpoint (HALLMARK Pathways) between Swab HI and BALF ICU. (A-D) NES: Normalized enrichment score. See also Table S6.

We next performed RNA-seq of the BALF of a subset of ICU-isolated patients and of subjects with noninfectious lung pathologies (Table S6). Gene set enrichment analysis confirmed that IFN responses characterize COVID-19 patients compared to non-microbially infected patients (Figures 5A and 5B). In keeping with the capacity of IFNs to increase apoptosis and facilitate lung tissue damage (Broggi et al., 2020a; Major et al., 2020), gene enrichment also revealed that the p53 pathway is significantly upregulated in COVID-19 patients (Figures 5A and 5C). Notably, the IFN landscape in the upper and lower airways of critical patients was strikingly similar (Figure 5D). Also, the induction of ISGs that protect against SARS-CoV2 was significantly decreased in the lower airways of critical COVID-19 patients compared with the upper airways of patients with milder, as well as similar, disease severity (Figures S5A–S5C). The gene signatures in the upper airways of mildly ill patients, compared with either the upper or lower airways of critical patients, were enriched for pathways associated with the induction of ISGs and other inflammatory pathways, (Figure 5E). In keeping with the capacity of IFNs to dampen cell proliferation and delay tissue repair (Broggi et al., 2020a; Major et al., 2020), gene programs linked to proliferation were significantly downmodulated in the lower airways of critical patients compared to the upper airways of subjects with a mild disease (Figure S5D).

Overall, these data demonstrate that a unique IFN signature characterizes severe-to-critical COVID-19 patients along the respiratory tract and that the induction of unique set of IFNs is coupled with the induction of either protective ISGs or gene programs associated with apoptosis and reduced proliferation.

### A unique protein IFN signature characterizes the lower airways of COVID-19 patients compared to patients with other ARDS or noninfectious lung pathologies

**Figure 6:**
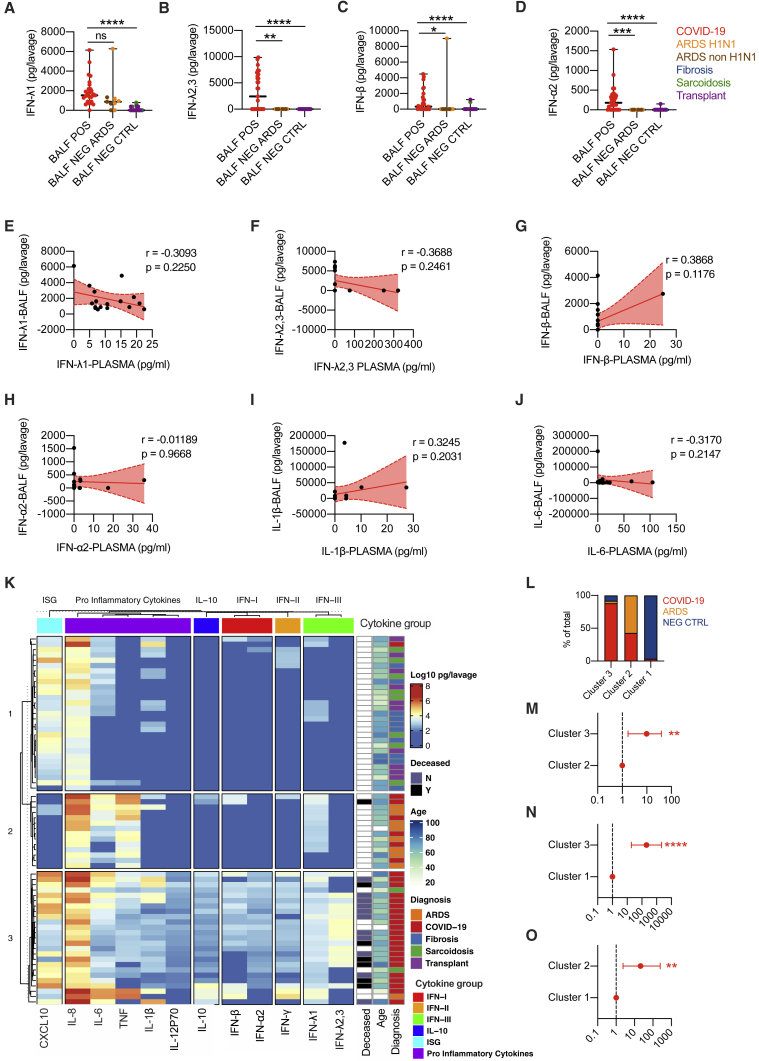
A unique protein IFN signature characterizes the lower airways of COVID-19 patients compared to patients with other ARDS or noninfectious lung pathologies (A–D) IFN-λ1 (A), IFN-λ2,3 (B), IFN-β (C), and IFN-α2 (D) protein levels were measured in the BALF of COVID-19 (BALF POS; 29, depicted with red dots), ARDS (BALF NEG ARDS; 5 were diagnosed H1N1 and are depicted with orange dots, and the remaining 4 are depicted with brown dots), non-microbially infected (BALF NEG CTRL; 10 affected by fibrosis are depicted with blue dots, 10 affected by sarcoidosis are depicted with green dots, and 10 transplant patients are depicted with purple dots). Each dot represents a patient. Median with range is depicted. (E–J) IFN-λ1 (E), IFN-λ2,3 (F), IFN-β (G), IFN-α2 (H), IL-1β (I), and IL-6 (J) protein levels in the BALF of COVID-19 patients (17) are plotted against protein levels in the plasma of the same patient. Each dot represents a patient. Linear regression lines (continuous line) and 95% confidence interval (dashed line and shaded area) are depicted in red. Spearman correlation coefficients (r) and p value (p) are indicated. (K) Heatmap comparison of IFN-α2, IFN-β, IFN-γ, IFN-λ1, IFN-λ2,3, IL-10, CXCL-10, IL-1β, IL-6, tumor necrosis factor (TNF), IL-8, and IL12p70 protein levels in the BALF of COVID-19 (29), ARDS (9), transplant (10), fibrosis (10), and sarcoidosis (10) patients. The color is proportional to the log10 transformed value of the amount of cytokine normalized for sample volume (picograms [pg]/lavage) of each cytokine. Rows in each group represent different patients. Unbiased K-means clustering was performed. Diagnosis, mortality, and age are annotated. (L) Percentage of patients with the indicated diagnosis within clusters identified in (K). (M–O) Odds ratio of containing COVID-19 patients in cluster 3 as compared to cluster 2 (M) and cluster 1 (N) and in cluster 2 as compared to cluster 1 (O) (clusters identified in J). Statistics by Kruskal-Wallis test with Dunn’s post hoc test: ns, not significant (p > 0.05); ^∗^p < 0.05; ^∗∗^p < 0.01; ^∗∗∗^p < 0.001; ^∗∗∗∗^p < 0.0001 (A–D) or chi-square test for odds ratio: ns, not significant (p > 0.05); ^∗^p < 0.05; ^∗∗^p < 0.01; ^∗∗∗^p < 0.001 (L–M). See also Figure S6 and Table S7.

**Figure S6:**
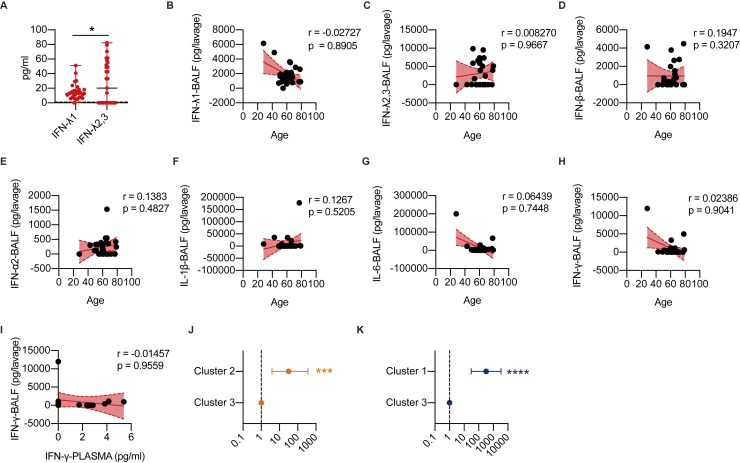
A unique protein IFN signature characterizes the lower airways of COVID-19 patients compared to patients with other ARDS or non-infectious lung pathologies, related to Figure 6 (A) IFN-λ1 and IFN-λ2,3 protein levels were measured in the BALF of COVID-19 patients (29). Each dot represents a patient. Median and range are depicted. Dashed line represents limit of detection. (B-H) IFN-λ1 (B), IFN-λ2,3 (C), IFN-β (D), IFN-α2 (E), IL-1β (F), IL-6 (G) and IFN-γ (H) protein levels in the BALF of COVID-19 patients (29) are plotted over age. (I) IFN-γ protein levels in the BALF of COVID-19 patients (17) are plotted against protein levels in the plasma. (J-K) Odds ratio of containing ARDS patients in Cluster 2 as compared to Cluster 3 (J) and of containing non-microbially infected control patients in Cluster 1 as compared to Cluster 3 (K) (Clusters identified in Figure 6J) (B-I) Each dot represents a patient. Linear regression lines (continuous line) and 95% confidence interval (dashed line and shaded area) are depicted in red. Spearman correlation coefficients (r) and p value (p) are indicated. Statistics: (A) Unpaired t test: ns, not significant (p > 0.05); ^∗^p < 0.05, ^∗∗^p < 0.01, ^∗∗∗^p < 0.001, and ^∗∗∗∗^p < 0.0001. (J-K) Chi Square test for odds ratio: ns, not significant (p > 0.05); ^∗^p < 0.05, ^∗∗^p < 0.01, ^∗∗∗^p < 0.001. See also Table S7.

Our data show unique patterns of IFN gene expression in the lower airways of severe COVID-19 patients. However, whether the relative distribution of the IFN members, as measured by mRNA transcripts, correlates with their protein levels remains unknown. We thus assessed protein levels of IFNs and other inflammatory cytokines in the BALF of subjects infected with COVID-19 compared to the BALFs of patients with ARDS not driven by SARS-CoV-2 or patients with noninfectious lung involvement including fibrosis, sarcoidosis, or lung transplant (hereafter referred to as “controls”) (Table S7). In keeping with results of the transcriptional analyses, the levels of IFN-III and IFN-I measured in BALF from patients with COVID-19 were elevated (Figures 6A–6D; STAR Methods) and, among IFN-III, showed a predominant induction of IFN-λ2,3 compared to IFN-λ1 (Figure S6A). IFN-III and IFN-I were also significantly upregulated in COVID-19 patients relative to controls and when compared to patients with ARDS of different etiologies (except for IFN-λ1) (Figure 6A–6D; STAR Methods). Also, we found no correlation between age and protein levels in the lower airways of severe COVID-19 patients (Figures S6B–S6H; STAR Methods).

When we compared the protein levels in the BALF and plasma of a subset of COVID-19 patients (STAR Methods), no correlation between these levels for any protein analyzed was found (Figures 6E–6J and S6I), confirming at the protein level the transcriptional differences recently highlighted between the peripheral blood and the lungs of COVID-19 patients (Overholt et al., 2021).

When we performed unbiased K-means clustering of the protein analyzed, we found that COVID-19 patients were significantly enriched in cluster 3, which is characterized by a unique signature of IFNs (which encompasses all three IFN families) and IL-10 production (Figures 6K–6O; STAR Methods). Many proinflammatory cytokines are also upregulated in cluster 2, which is enriched in patients who have ARDS that is not driven by SARS-CoV-2 (Figures 6L and S6J); most of these patients also express IFN-λ1, but not other IFNs. Control patients were, in contrast, enriched in cluster 1, characterized by low proinflammatory cytokine and IFN responses (Figures 6L and S6K).

Overall, these data demonstrate that COVID-19 patients are characterized by a unique IFN signature in the lower airways relative to patients with ARDS of different etiology.

### Epithelial and immune cells dictate the IFN landscape

**Figure 7:**
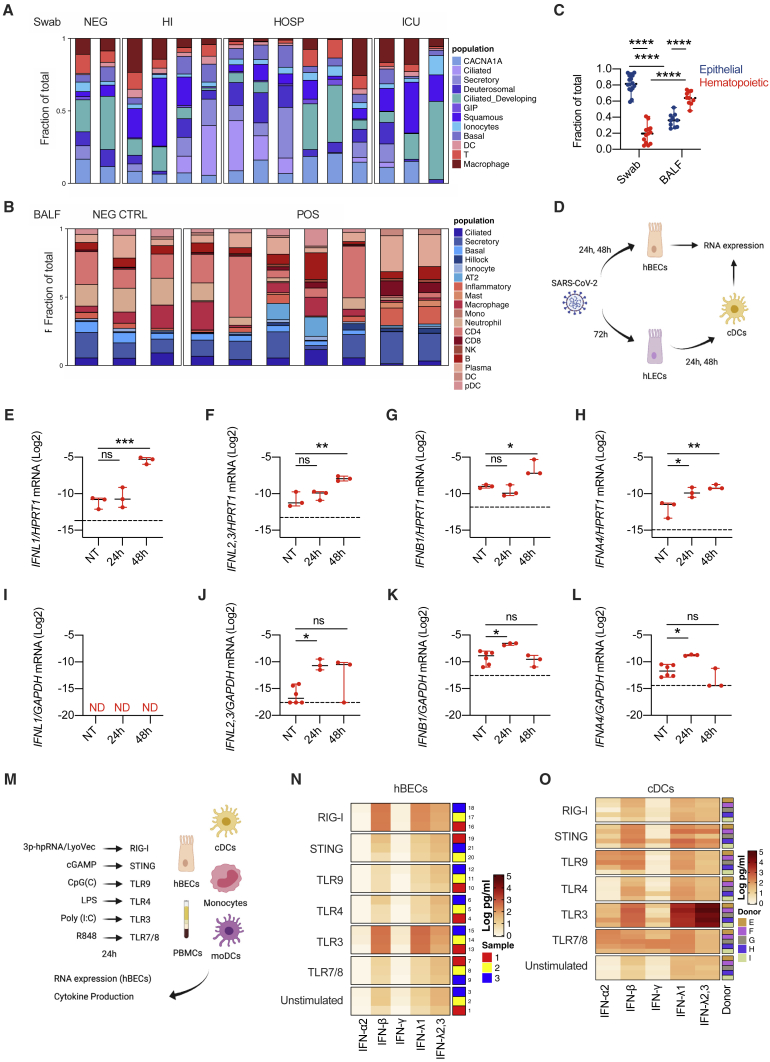
Epithelial and immune cells dictate the IFN landscape (A–C) Targeted RNA-seq of nasopharyngeal swabs from SARS-CoV-2-positive patients who were ICU inpatients (Swab ICU; 3), hospitalized (Swab HOSP; 6), or home-isolated (Swab HI; 4); SARS-CoV-2-negative (Swab NEG; 2) patients; and BALF from SARS-CoV-2-positive patients (BALF POS, 7) and patients with noninfectious lung pathologies (BALF NEG CTRL; 3) was performed. Data were deconvoluted based on publicly available single-cell RNA-seq (scRNA-seq) datasets (Ziegler et al., 2021) using CIBERSORTx (Newman et al., 2019) to extrapolate the relative cellular composition of samples. (A and B) Each cell population in swab (A) and BALF (B) samples is depicted as a fraction of total cells. (C) Fraction of epithelial or hematopoietic cells in swab and BALF samples is depicted. Each dot represents a patient. Median with range is depicted. (D) Schematic of experimental setup. hBECs were infected with SARS-CoV-2 for 24 and 48 h. hLECs were infected with SARS-CoV-2 for 72 h. cDCs were treated with supernatants from hLECs, infected or not, for 24 and 48 h. Gene expression was evaluated in hBECs and cDCs (created with BioRender). (E–H) *IFNL1* (E), *IFNL2,3* (F), *IFNB1* (G), and *IFNA4* (H) mRNA expression was evaluated in hBECs 24 and 48 h after infection with SARS-CoV-2. Each dot represents a biological replicate. Median with range is depicted. Dashed line represents limit of detection. (I–L) *IFNL1* (I), *IFNL2*,*3* (J), *IFNB1* (K), and *IFNA4* (L) mRNA expression was evaluated in cDCs 24 and 48 h after treatment with supernatants of uninfected or SARS-CoV-2-infected hLECs. Each dot represent a technical replicate. Median with range is depicted. Dashed line represents limit of detection. ND, not detected. (M) Schematic of experimental setup. hBECs, PBMCs, monocytes, cDCs, and moDCs were treated for 24 h with 3p-hpRNA/LyoVec, cGAMP, CpG(C), LPS, poly (I:C), or R848 for stimulation of RIG-I, STING, TLR9, TLR4, TLR3, or TLR7/8, respectively. Cytokine expression was evaluated on RNA extracted from cell lysates, and cytokine production was evaluated in supernatants (created with BioRender). (N–O) Heatmap representation of IFN-α2, IFN-β, IFN-γ, IFN-λ1, and IFN-λ2,3 production by hBECs (N) or cDCs (O) 24 h after treatment. The color is proportional to the log10-transformed concentration (pg/mL) of each cytokine. (N) Rows in each group represent a biological replicate. (O) Rows in each group represent different donors as depicted in the annotation. Expression is plotted as log2 (*gene*/*HPRT1* or *GAPDH* mRNA + 0.5 × gene-specific minimum) (E–L). Statistics by two-way ANOVA: ns, not significant (p > 0.05); ^∗^p < 0.05; ^∗∗^p < 0.01; ^∗∗∗^p < 0.001; ^∗∗∗∗^p < 0.0001 (C) or one-way ANOVA with Dunnett’s post hoc test: ns, not significant (p > 0.05); ^∗^p < 0.05; ^∗∗^p < 0.01; ^∗∗∗^p < 0.001; ^∗∗∗∗^p < 0.0001 (E–L). See also Figure S7 and STAR Methods.

**Figure S7:**
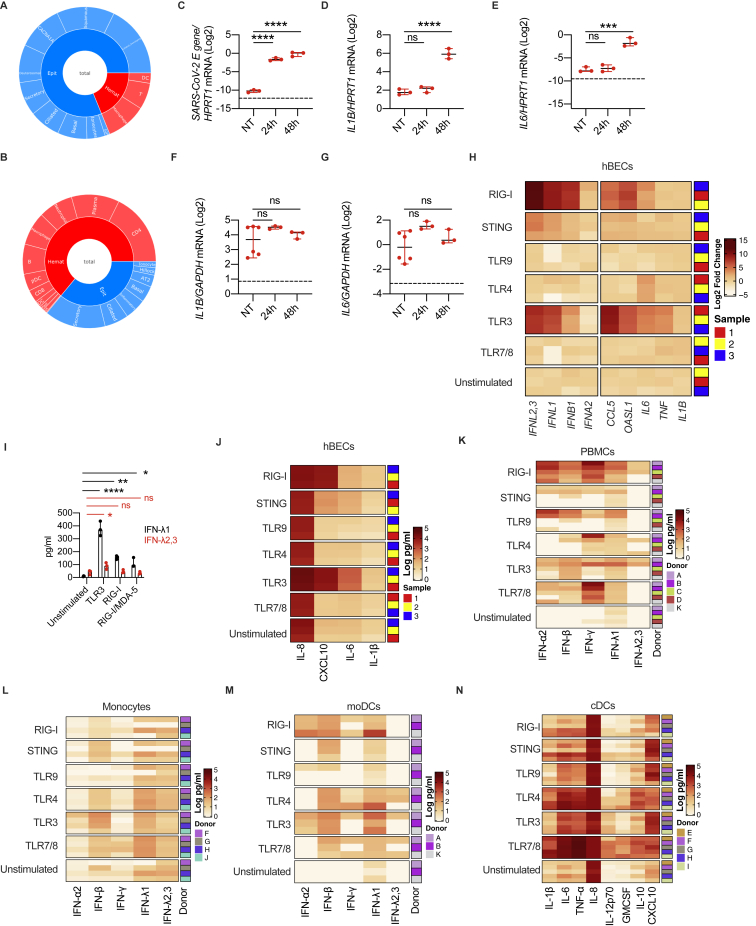
Epithelial and immune cells dictate the IFN landscape, related to Figure 7 (A-B) Sunburst plots representing cell population fractions in Swabs (A) and BALF (B) as identified in Figure 7A, B. (C-E) SARS-CoV-2 E gene (C), *IL1B* (D), *IL6* (E) mRNA expression was evaluated in hBECs 24 and 48 hours after infection with SARS-CoV-2. Each dot represents a biological replicate. Median with range is depicted. Dashed line represents limit of detection. (F-G) *IL1B* (F), *IL6* (G) mRNA expression was evaluated in cDCs 24 and 48 hours after treatment with supernatants of uninfected or SARS-CoV-2-infected hLECs. Each dot represents a biological replicate. Median with range is depicted. Dashed line represents limit of detection. (H-J) hBECs were treated with 3p-hpRNA/LyoVec, cGAMP, CpG(C), LPS, Poly (I:C) and R848 for stimulation of RIG-I, STING, TLR9, TLR4, TLR3 and TLR7/8 respectively. (H) Heatmap representation of *IFNL2*,*3*, *IFNL1*, *IFNB1*, *IFNA2*, *CCL5*, *OASL1*, *IL6*, *TNF* and *IL1B* mRNA expression 24 hours after treatment. The color is proportional to Log2 (Fold Change) of each gene. Rows in each group represent biological replicates distributed as indicated in the legend. (I) IFN-λ1 and IFN-λ2,3 production by hBECs treated for 24h with PRR ligands. Poly (I:C) (TLR3), 3p-hpRNA/LyoVec (RIG-I) and transfected Poly (I:C) (RIG-I/MDA5) were used. Each dot represents a biological replicate. Median with range is depicted. (J) Heatmap representation of IL-8, CXCL10, IL-6 and IL-1β production 24 hours after stimulation. The color is proportional to the Log10 transformed concentration (pg/ml) of each cytokine. Rows in each group represent a biological replicate. (K-M) Heatmap representation of IFN-α2, IFN-β, IFN-γ, IFN-λ1 and IFN-λ2,3 production by PMBCs (K), Monocytes (L), moDCs (M) 24 hours after treatment. The color is proportional to the Log10 transformed concentration (pg/ml) of each cytokine. (N) Heatmap representation of IL-1β, IL-6, TNF-α, IL-8, IL-12p70, GMCSF, IL-10 and CXCL10 production cDCs 24 hours after treatment. (J-N) The color is proportional to the Log10 transformed concentration (pg/ml) of each cytokine. Rows in each group represent different donors as depicted in the annotation on the right. (C-E, F, G) Expression is plotted as log2 (*gene*/*HPRT1* or *GAPDH* mRNA + 0.5 x gene-specific minimum). Statistics: (C-E, F,G, I) One-Way ANOVA with Dunnett’s post hoc test: ns, not significant (p > 0.05); ^∗^p < 0.05, ^∗∗^p < 0.01, ^∗∗∗^p < 0.001, and ^∗∗∗∗^p < 0.0001. See also STAR Methods.

Based on the heterogenous induction of IFNs along the respiratory tract of COVID-19 patients with different disease severity, we hypothesized that different populations of cells contribute to production of specific IFNs by activating discrete pattern recognition receptors (PRRs). Our finding that the mRNA for *IFNL1* is absent in the lower airways of COVID-19 patients (Figure 4A), but protein levels for IFN-λ1 are present at the same anatomical site (Figure 6A), suggests that cells that actively produce the mRNA for *IFNL1* are underrepresented in the BALF. However, *IFNL1* is one of the most upregulated genes in the upper airways, supporting the hypothesis that the cells that produce it are highly represented in the swabs. We thus explored the cellular composition of the swabs and BALF by deconvoluting our bulk RNA-seq data (Figures 7A–7C, S7A, and S7B). We found that the epithelial compartment, represented by several epithelial cell lineages, is more represented than the hematopoietic compartment in swabs from SARS-CoV-2-negative and positive subjects (Figures 7A, 7C, and S7A). In contrast, BALF from both SARS-CoV-2-negative and positive patients presents very diversified hematopoietic populations (Figures 7B and S7B) that are more represented than epithelial cells (Figure 7C).

We thus explored how epithelial cells, or phagocytes, differentially contribute to the production of IFNs during a SARS-CoV-2 encounter (Figure 7D). We confirmed that polarized hBECs of healthy individuals are sensitive to SARS-CoV-2 infection (Figure S7C) and respond by expressing IFNs (Figures 7E–7H) and proinflammatory cytokines (Figures S7D and S7E). Notably, hBECs infected with SARS-CoV-2 mostly produced IFN-λ1 compared to other IFNs (Figures 7E–7H). Among human phagocytes, plasmacytoid dendritic cells (pDCs) respond to SARS-CoV-2 by producing mainly IFN-I (Onodi et al., 2021). Based on the potent induction of IFN-III in patients with mild COVID-19, we focused our attention on conventional DCs (cDCs) that we recently described as major producers of IFN-III in the lungs of mice (Broggi et al., 2020a). Human cDCs isolated from the blood of healthy donors did not produce IFNs or other inflammatory cytokines when exposed to SARS-CoV-2 *in vitro* (data not shown). To test the possible involvement of cDCs during COVID-19, we infected a human lung epithelial cell (hLEC) line with SARS-CoV-2 and exposed cDCs to the supernatant of these cells. We found that only cDCs exposed to the supernatant of virally infected hLECs upregulated the expression of IFN-λ2,3 (but not IFN-λ1), members of the IFN-I family, as well as IL-1B and IL-6 (Figures 7I–7L, S7F, and S7G).

To identify the PRRs involved in the production of IFNs by either human epithelial cells or cDCs, we tested different PRR ligands (Figure 7M). In keeping with a central role of the RIG-I/MDA-5 pathway in epithelial cells for sensing SARS-CoV-2 (Liu et al., 2021; Wu et al., 2021; Yin et al., 2021), stimulation of the RIG-I pathway, and to a lesser extent of TLR3, in epithelial cells potently induced the transcripts of IFN-III and IFN-I, but not of other proinflammatory mediators (Figure S7H; STAR Methods). The analysis of protein levels confirmed the transcriptional data (Figures 7N, S7I, and S7J; STAR Methods). In keeping with SARS-CoV-2 infection, epithelial cells were more potent producers of IFN-λ1 compared to IFN-λ2,3 upon stimulation of TLR3, RIG-I and MDA-5 pathways (Figures 7N and S7I).

We next evaluated the response of cDCs. As a comparison, we also treated bulk peripheral blood mononuclear cells (PBMCs), monocytes isolated from PBMCs, and monocyte-derived DCs (moDCs). While PBMCs were particularly able to produce IFN-II in response to viral and bacterial ligands, cDCs were uniquely capable of producing very high levels of IFN-λ2,3 and, to a lesser extent, IFN-λ1, solely in response to TLR3 stimulation (Figures 7O and S7K–S7M; STAR Methods). Monocytes and moDCs were poor producers of IFNs in response to all the stimuli tested. When these analyses were extended to other inflammatory mediators, each cell type revealed a unique pattern of protein production (Figure S7N; STAR Methods), underscoring the complexity and cell specificity of the inflammatory response.

Collectively, these data demonstrate that epithelial cells preferentially produce IFN-λ1 upon SARS-CoV-2 infection and suggest that IFN production is driven via RIG-I/MDA-5 or TLR3 stimulation, that cDCs only respond to the supernatant of SARS-CoV-2-infected cells, and that TLR3 is the major driver of IFN-III production by human cDCs.

## Discussion

COVID-19 has caused millions of deaths and has had devastating societal and economic effects. Notwithstanding the efficacy of the COVID-19 vaccines, a better understanding of the molecular underpinnings that drive the severe disease in patients infected with the SARS-CoV-2 virus is imperative to implement effective additional prophylactic and/or therapeutic interventions. IFN-I and IFN-III are potent antiviral cytokines, and the potential of using clinical grade recombinant IFN-I or IFN-III as therapeutics has raised much hope and interest (Prokunina-Olsson et al., 2020). To date, though, opposing evidence has complicated our view of the role played by members of the IFN-I and IFN-III families during SARS-CoV-2 infection.

We found that in the upper airways of patients with mild manifestations, the presence of IFN-λ1 and IFN-λ3, but not IFN-λ2 or IFN-I, was associated with the induction of ISGs known to efficiently contain SARS-CoV-2. Our data also demonstrated that critically ill patients express high levels of IFN-I (and IFN-λ2) compared to subjects with mild disease or healthy controls. These patients show a reduced induction of protective ISGs and, in general, IFN responses. Two non-mutually exclusive explanations for this behavior may be that (1) the pattern of IFN expression of critically ill patients is less capable of inducing the protective ISGs; or (2) other factors, such as the production of specific antibodies that block ISG induction (Combes et al., 2021) or viral adaptation to evade control by IFN-I (Lei et al., 2020; Xia et al., 2020), restrain the capacity of this set of IFNs to mount a strong response.

The present in-depth analysis shows not only that high viral loads of SARS-CoV-2 induce the efficient production of IFN-III in the upper airways of younger and/or milder patients but also that severely ill COVID-19 patients are characterized by the highest levels of IFNs (at the mRNA as well as protein levels) in the lower airways. These data support the hypothesis that IFNs have opposing roles along the respiratory tract and reconcile some of the seemingly contradictory findings on IFNs in COVID-19 patients. Efficient initiation of IFN production in the upper airways can lead to a more rapid elimination of the virus and may limit viral spread to the lower airways, as suggested by studies that report defects in IFN signaling of severe COVID-19 patients (Bastard et al., 2020; Pairo-Castineira et al., 2021; Wang et al., 2021; Zhang et al., 2020). On the other hand, when the virus escapes immune control in the upper airways, the IFN production that is potently boosted in the lungs likely contributes to the cytokine storm and associated tissue damage that are typical of patients with severe-to-critical COVID-19, characterized by reduced proliferation and increased pro-apoptotic p53 transcriptional signatures.

Another novel finding in the present study is that the type of IFN produced in response to different PRR pathways varies according to cell types. In keeping with ACE2^+^ cells being the primary cells infected by SARS-CoV-2, we measured a potent immune response in hBECs, but not in cDCs, infected with SARS-CoV-2. Nevertheless, we found that cDCs efficiently express specific members of the IFN-III and IFN-I families when exposed to the supernatant of lung epithelial cells previously infected with SARS-CoV-2 or in response to double-stranded RNA (dsRNA). These data suggest that cDCs, despite not responding directly to SARS-CoV-2 infection, may play fundamental roles in recognizing intermediates of viral replication and/or damage-associated molecular patterns (DAMPs) released by infected cells that are dying.

Finally, our findings highlight the importance of the timing of production and/or administration of IFNs during COVID-19 and suggest that early administration (before infection or early after symptom onset) of specific recombinant IFN-III may be an effective therapeutic intervention and that targeting the upper airways, while avoiding systemic administration as previously proposed (Park and Iwasaki, 2020), represents the best way to exploit the antiviral activities of IFNs.

In conclusion, our data define the anatomical map of inter- and intra-family production of IFNs during COVID-19 and highlight how IFN production is linked to the different clinical outcomes, based on the location of the IFN response. Our findings reconcile a large portion of the literature on IFNs and further stress the key role played by IFN-III, compared to IFN-I, at mucosal surfaces during life-threatening viral infections. These findings will be fundamental for designing appropriate pharmacological interventions to prevent infection with SARS-CoV-2 or dampen the severity of COVID-19 and will help to better understand how the IFN landscape affects human immune responses to respiratory viral infections.

### Limitations of the study

Our findings shed new light on the nature of the IFNs and the molecular pathways that drive intrinsic immunity. The capacity of lung epithelial cells to recognize and respond to viral components is confounded by the presence of SARS-CoV-2 effector proteins that block immune recognition and IFN production (Banerjee et al., 2020; Konno et al., 2020; Lei et al., 2020; Wu et al., 2021). We show that high viral load in the upper airways of COVID-19 patients induces a potent immune response and that viral loads are not correlated per se with disease severity. High viral loads in the upper airways may therefore be associated with a protective immune response in young individuals while eliciting a dysregulated inflammatory response in older patients, as observed in our study. Nevertheless, additional studies are needed to directly link specific IFNs to particular cell types and, above all, specific protective or detrimental immune cell functions. As an example, our data suggest that cDCs do not directly sense SARS-CoV-2. Intriguingly, a recent report showed that specific cDC subtypes may instead directly respond to SARS-CoV-2 (Marongiu et al., 2021), but the capacity of these subtypes to produce specific IFNs remains an open question. Furthermore, understanding the specific contribution of different PRRs to the IFN response elicited in patients infected with SARS-CoV-2 also requires further analyses.

## STAR★Methods

### Key resources table

**Table:** 

REAGENT or RESOURCE	SOURCE	IDENTIFIER
**Antibodies**		

Mouse monoclonal anti-human CD14 (clone HCD14)	BioLegend	Cat#325621; RRID: AB_893252
Mouse monoclonal anti-human HLA-DR (clone L243)	BioLegend	Ca#307617; RRID: AB_493587
Mouse monoclonal anti-human CD11c (clone 3.9)	BioLegend	Cat#301607; RRID: AB_389350
Mouse monoclonal anti-human CD141 (clone M80)	BioLegend	Cat#344103; RRID: AB_1877220

**Bacterial and virus strains**		

SARS-CoV-2 Isolate England/02/2020	Respiratory Virus Unit, Public Health England, UK	GISAID accession number: EPI_ISL_407073
SARS-CoV-2 Isolate hCoV-19/Italy/UniSR1/2020	San Raffaele Hospital (Milan, Italy)	GISAID accession number: EPI_ISL_413489

**Biological samples**		

Nasopharyngeal swabs of 155 SARS-CoV-2 positive patients	San Raffaele Hospital (Milan, Italy)	N/A
Nasopharyngeal swabs of 28 SARS-CoV-2 negative patients	San Raffaele Hospital (Milan, Italy)	N/A
BALF of 26 SARS-CoV-2 positive patients	San Raffaele Hospital (Milan, Italy)	N/A
BALF of 26 SARS-CoV-2 positive patients	San Raffaele Hospital (Milan, Italy)	N/A
BALF of 29 SARS-CoV-2 positive patients	Luigi Sacco Hospital (Milan, Italy)	N/A
BALF of 63 SARS-CoV-2 negative patients (5 ARDS H1N1+, 4 ARDS H1N1-, 18 Fibrosis, 18 Sarcoidosis, 18 Transplant)	IRCCS Policlinico San Matteo Foundation (Pavia, Italy)	N/A
Blood collars from 11 healthy donors	Boston Children’s Hospital (Boston, MA USA)	N/A
Blood collar from 1 healthy donor	San Raffaele Hospital (Milan, Italy)	N/A

**Chemicals, peptides, and recombinant proteins**		

LPS	ENZO	Cat# ALX-581-013-L002
Poly (I:C) HMW	Invivogen	Cat# tlr-pic
R848	Invivogen	Cat# tlr-r848
CpG(C)	Invivogen	Cat# tlrl-2395
2′3′cGAMP	Invivogen	Cat# tlrl-nacga23-02
3p-hpRNA/LyoVec	Invivogen	Cat# tlrl-hprnalv
Lipofectamine 3000 Transfection Reagent	Invitrogen	Cat# L3000-008
Recombinant human IFN-λ1	Peprotech	Cat# 300-02L
Recombinant human IFN-λ2	Peprotech	Cat# 300-003K
Recombinant human IFN-λ3	R&D	Cat# 5259-IL
RPMI 1640 medium + GlutaMAX	GIBCO	Cat# 72400-047
Penicillin-Streptomycin	GIBCO	Cat# 15140122
FBS	GIBCO	Cat# 10437-028
DMEM	GIBCO	Cat# 11965-92
MEM non-essential amino acids solution	GIBCO	Cat# 11140050
HEPES buffer	GIBCO	Cat# 15630-080
MEM	GIBCO	Cat# 41090036
Histopaque	Sigma	Cat# 1077-1
GM-CSF	PeproTech	Cat# 300-03
IL-4	PeproTech	Cat# 200-04

**Critical commercial assays**		

FLOQSwabs	COPAN	Cat# 306C
Universal Transport Medium	COPAN	Cat# 306C
Cobas SARS-CoV-2 Test	Roche	Cat# P/N 09175431190
LEGENDplex	BioLegend	Cat# 740390
CD14 MicroBeads	Miltenyi Biotec	Cat# 130-050-201
CD141 (BDCA-3) MicroBead Kit	Miltenyi Biotec	Cat# 130-090-512
Pure Link RNA Micro Scale kit	Invitrogen	Cat# 12183016
SuperScript III First-Strand Synthesis System	Invitrogen	Cat# 18080051
Taqman Fast Advanced Master Mix	Applied Biosystems	Cat# 4444963
Power SYBR Green RNA-to-CT 1-step kit	Applied Biosystems	Cat# 4389986
SuperScript VILO cDNA Synthesis Kit	Invitrogen	Cat# 11754-05
Ion AmpliSeq Transcriptome Human Gene Expression Kit	Ion Torrent	Cat# A26325

**Deposited data**		

Targeted transcriptomics raw data	This study	GEO Series accession number:https://www.ncbi.nlm.nih.gov/geo/query/acc.cgi?acc=GSE182569
Targeted transcriptomics normalized read matrix	This study	Mendeley Data: https://doi.org/10.17632/pczgwbkfzk.1
qPCR gene expression matrix	This study	Mendeley Data: https://doi.org/10.17632/pczgwbkfzk.1
Cytokine expression matrix (BALF)	This study	Mendeley Data: https://doi.org/10.17632/pczgwbkfzk.1
Cytokine expression matrix (Plasma)	This study	Mendeley Data: https://doi.org/10.17632/pczgwbkfzk.1
Cytokine expression and gene expression matrix (hBECs)	This study	Mendeley Data: https://doi.org/10.17632/pczgwbkfzk.1
Cytokine expression matrix (Human Phagocytes)	This study	Mendeley Data: https://doi.org/10.17632/pczgwbkfzk.1
FACS plot of sorted and differentiated human phagocytes	This study	Mendeley Data: https://doi.org/10.17632/pczgwbkfzk.1

**Experimental models: Cell lines**		

NHBE	Lonza	Cat# CC-2540
Vero C1008 (Vero 76, clone E6, Vero E6)	ATCC	Cat# CRL-1586
Calu-3	ATCC	Cat# HTB-55

**Oligonucleotides**		

*IFNL1* Taqman Gene Expression Assay	Thermo Fisher	Cat# Hs01050642_gH
*IFNL2*,*3* Taqman Gene Expression Assay	Thermo Fisher	Cat# Hs04193047_gH
*IFNL4* Taqman Gene Expression Assay	Thermo Fisher	Cat# Hs04400217_g1
*IFNB1* Taqman Gene Expression Assay	Thermo Fisher	Cat# Hs01077958_s1
*IFNA2* Taqman Gene Expression Assay	Thermo Fisher	Cat# Hs00265051_s1
*IFNA4* Taqman Gene Expression Assay	Thermo Fisher	Cat# Hs01681284_sH
*IL1B* Taqman Gene Expression Assay	Thermo Fisher	Cat# Hs01555410_m1
*IL6* Taqman Gene Expression Assay	Thermo Fisher	Cat# Hs00174131_m1
*GAPDH* Taqman Gene Expression Assay	Thermo Fisher	Cat# Hs99999905_m1
*HPRT1* Taqman Gene Expression Assay	Thermo Fisher	Cat# Hs99999909_m1
*RSAD2* forward primer (GCTCTAAGAGAAGCAGAAAG)	Sigma	N/A
*RSAD2* reverse primer (CATCTTCTGGTTAGATTCAGG)	Sigma	N/A
*IFIT3* forward primer (ATGAGTGAGGTCACCAAG)	Sigma	N/A
*IFIT3* reverse primer (CCTTGAATAAGTTCCAGGTG)	Sigma	N/A
*LY6E* forward primer (CATTGGGAATCTCGTGAC)	Sigma	N/A
*LY6E* reverse primer (CACTGAAATTGCACAGAAAG)	Sigma	N/A
*APOL2* forward primer (GAGAGCAGTATCTTTATTGAGG)	Sigma	N/A
*APOL2* reverse primer (CAGTTGTAGCAGATTCTCTC)	Sigma	N/A
*UBC* forward primer (CGTCACTTGACAATGCAG)	Sigma	N/A
*UBC* reverse primer (TGTTTTCCAGCAAAGATCAG)	Sigma	N/A
SARS-CoV-2 E gene forward primer (ACAGGTACGTTAATAGTTAATAGCGT)	Tib-Molbiol	N/A
SARS-CoV-2 E gene probe (FAM-ACACTA GCCATCCTTACTGCGCTTCG-BBQ) (FAM: 6-carboxyfluorescein; BBQ: blackberry quencher)	Tib-Molbiol	N/A
SARS-CoV-2 E gene reverse primer (ATATTGCAGCAGTACGCACACA)	Tib-Molbiol	N/A

**Software and algorithms**		

Transcriptome Analysis Console (TAC) software with ampliSeqRNA plugin	ThermoFisher	N/A
CIBERSORTx	Newman et al., 2019	N/A
Fast Gene Set Enrichment Analysis package (fGSEA)	Korotkevich et al., 2021	N/A
ComplexHeatmap package	Gu et al., 2016	N/A

### Resource availability

#### Lead contact

Further information and requests for resources and reagents should be directed to and will be fulfilled by the lead contact, Ivan Zanoni (ivan.zanoni@childrens.harvard.edu).

#### Materials availability

This study did not generate new unique reagents.

### Experimental model and subject details

#### Clinical samples for gene expression analysis and targeted RNA-sequencing

Nasopharyngeal swabs were collected using FLOQSwabs® (COPAN Cat#306C) in UTM® Universal Transport Medium (COPAN Cat#306C) from 155 SARS-CoV-2 positive patients and from 28 negative subjects undergoing screening for suspected social contacts with SARS-CoV-2 positive subjects. Nasopharyngeal swabs were collected at San Raffaele Hospital (Milan, Italy) from April to December 2020. BALF was obtained from 26 SARS-CoV-2-positive patients hospitalized at San Raffaele Hospital (Milan, Italy) from March to May 2020. BALF was obtained from 24 non-infected patients: lung fibrosis patients (8) were collected from May 2018 to September 2020; sarcoidosis patients (8) were collected from August to July 2020; lung transplant patients (8) were collected from January 2018 to September 2020 by IRCCS Policlinico San Matteo Foundation (Pavia, Italy). See Tables S1–S6 for patient information. All samples were stored at −80°C until processing. 500 μL of each BALF and swab sample were lysed and used for RNA extraction (see *RNA extraction protocol and Real-Time PCR for clinical samples and hBECs*).

Clinical metadata were obtained from the COVID-BioB clinical database of the IRCCS San Raffaele Hospital. The study was approved by the Ethics Committee of San Raffaele Hospital (protocol 34/int/2020). All of these patients signed an informed consent form. Our research was in compliance to the Declaration of Helsinki.

#### Clinical samples for cytokine quantification in BALF and plasma

BALF from 29 SARS-CoV-2 positive patients hospitalized in the Intensive Care Unit (ICU) at Luigi Sacco Hospital (Milan, Italy) were collected from September to November 2020. The total volume for each lavage was 120ml. Blood from 17 of these patients was also collected on the same day. BALF from patients affected by ARDS (9 in total, 5 of which were diagnosed H1N1 influenza A virus) were collected from February 2014 to March 2018. Samples from: lung fibrosis patients (10) were collected from May 2018 to September 2020; sarcoidosis patients (10) were collected from August to July 2020; lung transplant patients (10) were collected from January 2018 to September 2020 by IRCCS Policlinico San Matteo Foundation (Pavia, Italy). The total volume for each lavage was 150ml. None of the patients affected by lung fibrosis, sarcoidosis or that received lung transplant was diagnosed a respiratory viral or bacterial infection. See Table S7 for patient information.

Research and data collection protocols were approved by the Institutional Review Boards (Comitato Etico di Area 1) (protocol 20100005334) and by IRCCS Policlinico San Matteo Foundation Hospital (protocol 20200046007). All patients signed an informed consent form. Our research was in compliance to the Declaration of Helsinki.

#### Isolation of human phagocytes

Human phagocytes were isolated from collars of blood received from Boston Children’s Hospital blood donor center for *in vitro* stimulations and from San Raffaele Hospital blood donor center for SARS-CoV-2 infections. Briefly, blood was diluted 1:2 in PBS and PBMCs were isolated using a Histopaque (Sigma Cat# 1077-1) gradient. Monocytes were positively selected from PBMCs with CD14 MicroBeads (Miltenyi Biotec Cat# 130-050-201) by MACS technology. MoDCs were differentiated from monocytes in the presence of GM-CSF 20ng/ml (PeproTech Cat# 300-03) and IL-4 20ng/ml (PeproTech Cat# 200-04) for 7 days. MoDCs differentiation was tested for CD14 downregulation and HLA-DR expression. cDCs were positively selected from PBMCs with CD141 (BDCA-3) MicroBead Kit (Miltenyi Biotec Cat# 130-090-512) by MACS technology. Purity and differentiation were assessed by FACS and representative plots are available on Mendeley (see Key resources table)

hBECs were expanded in a T-75 flask to 60% confluence and then trypsinized and seeded either on 48 well plates (2x10^5^ cells/well) for IFN stimulations or (3x10^4^ cells/transwell) onto 0.4 μm pore size clear polyester membranes (Corning Cat# 3470) coated with a collagen solution for PRR agonist stimulations and SARS-CoV-2 infections.

#### SARS-CoV-2 propagation and titration

For hBECs infection experiments with SARS-CoV-2, the isolate England/02/2020 (GISAID accession ID: EPI_ISL_407073) was propagated and titrated in Vero E6 cells (ATCC Cat# CRL-1586). For cDCs infection experiments with SARS-CoV-2 the isolate hCoV-19/Italy/UniSR1/2020 (GISAID accession ID: EPI_ISL_413489) was propagated and titrated in Vero E6 cells (ATCC Cat# CRL-1586). All infection experiments were performed in a biosafety level-3 (BSL-3) laboratory.

#### Evaluation of SARS-CoV-2 RNA amount in clinical samples

The viral load was inferred on nasopharyngeal swabs through cycle threshold (Ct) determination with Cobas® SARS-CoV-2 Test (Roche Cat# P/N 09175431190), a real-time PCR dual assay targeting ORF-1a/b and E-gene regions on SARS-CoV-2 genome. The mean between ORF-1a/b and E Ct was used as an indirect measure of the viral load. Non-infectious plasmid DNA containing a specific SARS-CoV-2 sequence and a pan-Sarbecovirus sequence is used in the test as positive control. A non-Sarbecovirus related RNA construct is used as internal control. The test is designed to be performed on the automated Cobas® 6800 Systems under Emergency Use Authorization (EUA). The test is available as a CE-IVD test for countries accepting the CE-mark.

#### Culture of primary NHBE (hBECs) and Calu3 cells (hLECs)

NHBE (hBECs) were expanded in a T-75 flask to 60% confluence and then trypsinized and seeded either on 48 well plates (2x10^5^ cells/well) for IFN stimulations or (3x10^4^ cells/transwell) onto 0.4 μm pore size clear polyester membranes (Corning Cat# 3470) coated with a collagen solution for PRR agonist stimulations and SARS-CoV-2 infections.

Calu-3 (hLECs, ATCC Cat# HTB-55) were cultured in MEM (GIBCO Cat# 41090036) and supplemented with MEM non-essential amino acids solution (GIBCO Cat#11140050), Penicillin-Streptomycin (GIBCO Cat#11140050), Sodium Pyruvate and 10% FBS (GIBCO Cat#10437-028).

### Method details

#### *In vitro* stimulation and SARS-CoV-2 infection of hBECs

IFN stimulations were performed one day after seeding by treating cells with 2ng/ml IFN-λ1, IFN-λ2 and IFN-λ3 for 4 and 24 hours. Cell lysates were processed for RNA extraction as described below. For PRR agonist stimulations and SARS-CoV-2 infections cells were grown in submersion until confluent, and then exposed to air to establish an air-liquid interface (ALI). At ALI day 15, cells were stimulated with LPS (100 ng/ml), R848 (10 μg/ml), CpG(C) (1 μM), Poly (I:C) (50 μg/ml), Poly (I:C) (1 μg/10^6^ cells) + Lipofectamine, 3p-hpRNA/LyoVec (100 ng/ml), and cGAMP (10 μg /ml). Supernatants and cell lysates were collected 24 hours post treatment. Supernatants were processed with LEGENDplex™ (BioLegend Cat# 740390) according to manufacturer’s instructions and read by flow cytometry. Lysates were processed for RNA extraction as described below. For SARS-CoV-2 infections on day 15 of ALI cells were washed apically with PBS and infected at a multiplicity of infection (MOI) of 10^−1^ for 30 minutes at 37°C. The inoculum was then removed, and cell lysates were collected at 24 or 48 hours post infection for RNA extraction as described below.

#### Measurement of cytokine levels on BALF and plasma samples

BALF specimens from COVID-19 patients were managed in a biosafety level 3 laboratory until viral inactivation with a 0.2% SDS and 0.1% Tween-20 solution and heating at 65 °C for 15 min. Cell-free BALF supernatants were stored at − 20 °C until analysis. Blood was centrifuged at 400 g for 10 minutes without brake and plasma was stored at − 20 °C until analysis. Samples were processed with LEGENDplex™ (BioLegend Cat# 740390) according to manufacturer’s instructions and read by flow cytometry.

#### *In vitro* stimulation of human phagocytes with PRR agonists and supernatant from SARS-CoV-2-infected hLECs

PBMCs, monocytes, moDCs and cDCs were stimulated with LPS (100 ng/ml), R848 (10 μg/ml), CpG(C) (1 μM), Poly (I:C) (50 μg/ml), 3p-hpRNA/LyoVec (2.5 μg/ml), and cGAMP (10 μg/ml). Supernatants were collected 24 hours post treatment and stored at − 20 °C until analysis. cDCs were also stimulated with conditioned media from hLECs. hLECs were infected or not with SARS-CoV-2 at an MOI of 10^−1^ and supernatant was collected 72 hours post infection. Cell lysates were collected 24 and 48 hours after treatment for RNA extraction as described below.

#### RNA extraction protocol and Real-Time PCR from clinical samples and hBECs

RNA was extracted from nasopharyngeal swabs, BALFs, hBECs (stimulated with PRR agonists, with IFNs and infected with SARS-CoV-2) lysates and cDCs (stimulated with supernatant from SARS-CoV-2 infected hLECs) using Pure Link RNA Micro Scale kit (Invitrogen Cat# 12183016) according to manufacturer’s instruction, including in-column DNase treatment. Reverse transcription was performed on all samples except IFN-treated hBECs using SuperScript™ III First-Strand Synthesis System (Invitrogen Cat# 18080051) according to manufacturer’s instruction. qRT-PCR analysis was then carried out with Taqman™ Fast Advanced Master Mix (Applied Biosystems Cat#4444963) by using specific Taqman™ Gene Expression Assays from Thermo Fisher. *IFNL1 (*Hs01050642_gH), *IFNL2*,*3 (*Hs04193047_gH), *IFNL4* (Hs04400217_g1), *IFNB1* (Hs01077958_s1), *IFNA2* (Hs00265051_s1), *IFNA4* (Hs01681284_sH), *IL1B* (Hs01555410_m1) and *IL6* (Hs00174131_m1) expression was assessed with respect to the housekeeping gene *GAPDH* (Hs99999905_m1) or *HPRT1* (Hs99999909_m1). qRT-PCR was performed on IFN-treated hBECs with Power SYBR Green RNA-to-CT 1-step kit (Applied Biosystems Cat#4389986) from Thermo Fisher using primers (Sigma) for the following genes: *UBC*, *RSAD2*, *IFIT3*, *LY6E*, *APOL2*. Expression was assessed with respect to the housekeeping *UBC*. SARS-CoV-2 E gene expression in infected hBECs was quantified by real-time reverse transcription PCR. All transcripts were tested in triplicate for each sample on ViiA7 Real-Time PCR System (Thermo Fisher) for clinical samples, on Quantastudio 3 Real-Time PCR System (Thermo Fisher) for hBECs stimulated with PRR agonists and infected with SARS-CoV-2 and on CFX384 real time cycler (Bio-rad) for hBECs stimulated with IFNs and cDCs infected with SARS-CoV-2.

#### Targeted Transcriptomics

For targeted transcriptome sequencing, RNA (15ng) isolated from clinical samples described in Tables S4 and S6 was retro-transcribed to cDNA using SuperScript VILO cDNA Synthesis Kit (Invitrogen Cat# 11754-05). Barcoded libraries were prepared using the Ion AmpliSeq Transcriptome Human Gene Expression Kit (Ion Torrent Cat# A26325) as per the manufacturer’s protocol and sequenced using an Ion S5 system (Ion Torrent Cat# A27212). Differential gene expression analysis was performed using the Transcriptome Analysis Console (TAC) software with the ampliSeqRNA plugin (ThermoFisher Scientific).

We used CIBERSORTx (Newman et al., 2019) to estimate the abundances of epithelial end hematopoietic cell types using using bulk gene expression data as an input and scRNaseq signature matrices from single-cell RNA sequencing data to provide the reference gene expression profiles of pure cell populations. The scRNaseq signature matrix used to deconvolute RNaseq dataset from swabs or BALFs were derived from Wauters et al. (2021) and Ziegler et al. (2021).

Gene set enrichment analysis and enrichment plot were generated in R using the Fast Gene Set Enrichment Analysis package (fGSEA) (Korotkevich et al., 2021). Heatmaps were generated in R and visualized with the ComplexHeatmap package (Gu et al., 2016). Clustering analysis was performed using Euclidean distances on individual z-scores. Code available upon request.

### Quantification and statistical analysis

One-way ANOVA with Turkey’s post hoc test was used to compare continuous variables among multiple groups. Kruskal-Wallis test with Dunn’s post hoc test or Multiple Mann-Whitney tests with Holm-Šídák method were used instead when data did not meet the normality assumption. Fisher’s exact test was used to compare categorical variables. Spearman correlation analysis was used to examine the degree of association between two continuous variables. To establish the appropriate test, normal distribution and variance similarity were assessed with the D’Agostino-Pearson omnibus normality test.

Cluster analysis with unbiased K-mean methods based on the expression of IFN-I, IFN-III and the proinflammatory cytokine IL-1β were used to classify a subset of COVID-19 patients into 3 exclusive clusters.

Cluster analysis with unbiased K-mean methods based on the expression of Interferons and pro-inflammatory cytokines in the BALF were used to classify COVID-19 patients, non-COVID-19 ARDS patients, and controls into 3 exclusive clusters. Heatmaps and K-mean clustering were generated in R and visualized with the ComplexHeatmap package. Clustering analysis was performed using Euclidean distances. Estimated (K) value was selected based on the elbow point cluster number. Logistic regression models were performed to estimate the association of gene expression as binary outcome within viral load terciles (defined by mean viral RNA CT < 20, > 20 and < 30, > 30), and clusters (cluster 1, cluster 2 and cluster 3). Interaction between viral load terciles and age groups (≥70 years versus < 70 years) were tested to detect significant difference between elder patients and young patients in their gene expression response to different levels of viral load. All statistical analyses were two-sided and performed using Prism9 (Graphpad) software or SAS version 9.4 (SAS Institute). All statistical analyses are indicated in figure legends. Throughout the paper significant is defined as follows: ns, not significant (p > 0.05); ^∗^p < 0.05, ^∗∗^p < 0.01, ^∗∗∗^p < 0.001, and ^∗∗∗∗^p < 0.0001.

### Additional resources

A subset of samples included in this study were obtained from the following clinical trial: NCT04318366, https://www.clinicaltrials.gov/ct2/show/NCT04318366.

## Data Availability

• Targeted transcriptomics data have been deposited at GEO and are publicly available as of the date of publication. Accession numbers are listed in the Key resources table. • Gene expression matrix from targeted transcriptomics, Gene expression matrix from qPCR experiments, cytokine expression matrix from multiplex analysis of BALF, Plasma and supernatants of phagocytes are deposited at Mendeley and are publicly available as of the date of publication. The DOI is listed in the Key resources table. • The code used to analyze the data is available upon request to the corresponding authors.
