# Supplementary material for: The interferon landscape along the respiratory tract impacts the severity of COVID-19
Source: Cell. 2021 Sep 16;184(19):4953–4968.e16. doi: 10.1016/j.cell.2021.08.016 (PMC8373821; doi:10.1016/j.cell.2021.08.016)
Supplement: Document S1. Tables S1–S7 [file mmc1.pdf]

Cell, Volume *184*

Supplemental information

The interferon landscape along the respiratory tract impacts the severity of COVID-19

Benedetta Sposito, Achille Broggi, Laura Pandolfi, Stefania Crotta, Nicola Clementi, Roberto Ferrarese, Sofia Sisti, Elena Criscuolo, Roberto Spreafico, Jaclyn M. Long, Alessandro Ambrosi, Enju Liu, Vanessa Frangipane, Laura Saracino, Sara Bozzini, Laura Marongiu, Fabio A. Facchini, Andrea Bottazzi, Tommaso Fossali, Riccardo Colombo, Massimo Clementi, Elena Tagliabue, Janet Chou, Antonio E. Pontiroli, Federica Meloni, Andreas Wack, Nicasio Mancini, and Ivan Zanoni

		Swab NEG	Swab POS total	Swab POS +	Swab POS ++	Swab POS +++
	**Samples (#)**	28(183)	155(183)	51(155)	46(155)	58(155)
**Age**	**Minimum**	23	10	17	10	26
	**Maximum**	86	98	93	98	97
	**Q1**	46	44	46	46	48
	**Q3**	72	80	83	80	79
	**IQR**	26	36	37	34	31
	**Median**	56	58	68	55	61
	**Mean**	57	60	62	58	62
	**≥ 70 (#)**	8(28)	61(155)	25(51)	16(46)	20(58)
	**< 70 (#)**	20(28)	94(155)	26(51)	30(46)	38(58)
	**≥ 70 (%)**	29	39	49	35	34
	**< 70 (%)**	71	61	51	65	66
**Sex**	**F (#)**	15(28)	85(155)	27(51)	27(46)	31(58)
	**M (#)**	13(28)	70(155)	24(51)	19(46)	27(58)
	**F (%)**	54	55	53	59	53
	**M (%)**	46	45	47	41	47

SUPPLEMENTAL TABLE 1. Patient information for Swab NEG and Swab POS samples used for gene expression. Related to FIGURE and SUPPLEMENTAL FIGURE 1. Age, sex and severity characteristics of patient cohorts analyzed in **Figure 1 and Supplemental Figure 1**. Nasopharyngeal swabs from SARS-CoV-2-negative (Swab NEG) and -positive (Swab POS total) subjects, that were further divided in viral load terciles (“Swab POS +”, “Swab POS++”, “Swab POS+++”), were analyzed by qPCR. Q1=quartile 1, Q3=quartile 3, IQR=interquartile range, ≥ 70=over or equal to 70 years old, <70=under 70 years old, F=female, M=male, #=number of samples, %=percentage of samples.

		Age <70		Age ≥70		
**Outcome/gene**	**Viral load tercile**	**Odds ratio**	**P value**	**Odds ratio**	**P value**	**P value for interaction**
IFNA2	+	Reference(1.0)		Reference(1.0)		0.38
	++	0.7(0.3,2.2)	0.59	0.5(0.1,2.1)	0.36	
	+++	3.2(1.1,9.2)	0.03	0.9(0.2,3.6)	0.89	
IFNA4	+	Reference(1.0)		Reference(1.0)		0.03
	++	1.1(0.2,5.5)	0.92	0.5(0.1,2.4)	0.38	
	+++	10.7(2.4,47.9)	0.002	0.8(0.2,3.0)	0.70	
IFNB1	+	Reference(1.0)		Reference(1.0)		0.14
	++	0.5(0.1,1.5)	0.19	0.5(0.1,1.8)	0.29	
	+++	8.8(2.6,30.2)	<0.001	1.7(0.4,6.8)	0.49	
IFNL1	+	NE		NE		0.06
	++	Reference(1.0)		Reference(1.0)		
	+++	48.0(5.8,399.0)	<0.001	2.1(0.5,8.9)	0.33	
IFNL2,3	+	Reference(1.0)		Reference(1.0)		<0.001
	++	1.0(0.3,3.9)	0.96	0.8(0.2,3.0)	0.80	
	+++	22.1(6.0,82.0)	<0.001	1.0(0.3,3.5)	0.95	
IFNL4	+	Reference(1.0)		Reference(1.0)		0.13
	++	NE		0.6(0.1,3.2)	0.47	
	+++	4.2(1.0,17.6)	0.049	0.6(0.2,2.1)	0.39	
IL1B	+	Reference(1.0)		Reference(1.0)		0.13
	++	5.3(1.6,17.6)	0.005	0.8(0.2,3.6)	0.73	
	+++	11.5(3.1,42.6)	<0.001	NE		
IL6	+	Reference(1.0)		Reference(1.0)		0.80
	++	2.7(0.3,27.8)	0.40	7.8(0.8,79.3)	0.08	
	+++	28.1(3.4,233.1)	0.002	83.90(8.2,857.3)	<0.001	

SUPPLEMENTAL TABLE 2. Odds ratio of expressing/not each gene across viral load terciles and age groups in swabs from SARS-CoV-2 positive patients. Related to FIGURE SUPPLEMENTAL 1. Odds ratio of expressing *IFNL1* in "+++" with respect to "++" *and IFNL2,3*, *IFNL4*, *IFNB1*, *IFNA2*, *IFNA4*, *IL1B*, and *IL6* mRNA in "+++" and "++" with respect to "+" swabs from SARS-CoV-2 positive ≥ 70 and < 70 patients was calculated. Odds ratio column indicates the odds ratio and associated 95% confidence interval in brackets. P value column indicates the associated P value for each cohort of patients. Interaction between viral load terciles and age groups (≥70 years vs <70 years) was tested and P values for interaction are indicated. NE=not estimable.

		Gene Expression			Mean Viral RNA CT progression		
		**HI**	**HOSP**	**ICU**	**HI**	**HOSP**	**ICU**
	**Samples (#)**	9(31)	19(31)	3(31)	6(12)	5(12)	1(12)
**Age**	**Minimum**	22	38	60	50	32	NA
	**Maximum**	78	97	69	84	84	NA
	**Q1**	42	57	64	54	51	NA
	**Q3**	51	85	69	73	81	NA
	**IQR**	9	29	5	19	30	NA
	**Median**	47	76	68	68	73	91
	**Mean**	50	71	66	66	64	91
**Sex**	**F (#)**	4(9)	9(19)	1(3)	3(6)	2(5)	1(1)
	**M (#)**	5(9)	10(19)	2(3)	3(6)	3(5)	0(1)
	**F (%)**	44	47	33	50	40	100
	**M (%)**	56	53	67	50	60	0

SUPPLEMENTAL TABLE 3. Patient information for HI, HOSP and ICU Swab samples for gene expression and for Mean Viral RNA CT progression analysis. Related to FIGURE 2 and SUPPLEMENTAL FIGURE 2. Age, sex and severity characteristics of patient cohorts analyzed in **Figure 2 and Supplemental Figure 2**. Nasopharyngeal swabs from SARS-CoV-2 positive subjects that were either home-isolated (HI), hospitalized (HOSP)) or hospitalized and admitted to the ICU (ICU) were analyzed by qPCR for gene expression or were analyzed longitudinally for mean viral RNA CT. Q1=quartile 1, Q3=quartile 3, IQR=interquartile range, F=female, M=male, #=number of samples, %=percentage of samples.

		Swab NEG	Swab POS
	Samples (#)	3(18)	15(18)
Viral load tercile	+ (#)	NA	3(15)
	++ (#)	NA	6(15)
	+++ (#)	NA	6(15)
Severity	HI (#)	NA	5(15)
	HOSP (#)	NA	7(15)
	ICU (#)	NA	3(15)
Age	Minimum	29	42
	Maximum	86	96
	Q1	51	51
	Q3	80	77
	IQR	29	27
	Median	73	68
	Mean	63	65
Sex	F (#)	1(3)	6(15)
	M (#)	2(3)	9(15)
	F (%)	33	40
	M (%)	67	60

SUPPLEMENTAL TABLE 4. Patient information for swab samples used for RNA-Seq. Related to FIGURE and SUPPLEMENTAL FIGURE 3. Age and sex characteristics of patient cohorts analyzed in **Figure 3 and Supplemental Figure 3**. Nasopharyngeal swabs from SARS-CoV-2 positive patients divided in viral load terciles ("+++", "++", "+") and that were either home-isolated (HI), hospitalized (HOSP)) or hospitalized and admitted to the ICU (ICU) were analyzed by RNA-Seq. Q1=quartile 1, Q3=quartile 3, IQR=interquartile range, ≥ 70=over or equal to 70 years old, <70=under 70 years old, F=female, M=male, #=number of samples, %=percentage of samples.

		BALF NEG CTRL	BALF POS	Swab POS
	Samples (#)	24(72)	26(72)	22(72)
Severity	HOSP (#)	NA	5(26)	19(22)
	ICU (#)	NA	21(26)	3(22)
Diagnosis	Fibrosis (#)	8(24)	NA	NA
	Sarcoidosis (#)	8(24)	NA	NA
	Transplant (#)	8(24)	NA	NA
Age	Minimum	33	47	38
	Maximum	100	86	97
	Q1	50	61	58
	Q3	64	71	84
	IQR	14	10	27
	Median	57	66	69.5
	Mean	59	65	70
Sex	F (#)	12(24)	4(26)	12(22)
	M (#)	12(24)	22(26)	10(22)
	F (%)	50	15	55
	M (%)	50	85	45

SUPPLEMENTAL TABLE 5. Patient information for swab and BALF samples used for gene expression. Related to FIGURE and SUPPLEMENTAL FIGURE 4. Age and sex characteristics of patient cohorts analyzed in **Figure 4 and Supplemental Figure 4**. Nasopharyngeal swabs and BALF from SARS-CoV-2 positive patients that were either home-isolated (HI), hospitalized (HOSP)) or hospitalized and admitted to the ICU (ICU) and BALF from SARS-CoV-2 negative patients were analyzed by qPCR. Q1=quartile 1, Q3=quartile 3, IQR=interquartile range, , F=female, M=male, #=number of samples, %=percentage of samples.

		BALF NEG CTRL	BALF POS	Swab POS
	Samples (#)	5(27)	7(27)	15(27)
Viral load tercile	+ (#)	NA	NA	3(15)
	++ (#)	NA	NA	6(15)
	+++ (#)	NA	NA	6(15)
Diagnosis	Fibrosis (#)	1(5)	NA	NA
	Sarcoidosis (#)	2(5)	NA	NA
	Transplant (#)	2(5)	NA	NA
Severity	HI (#)	NA	0(7)	5(15)
	HOSP (#)	NA	0(7)	7(15)
	ICU (#)	NA	7(7)	3(15)
Age	Minimum	45	48	42
	Maximum	64	72	96
	Q1	53	66	51
	Q3	63	71	77
	IQR	10	5	27
	Median	63	69	68
	Mean	58	66	65
Sex	F (#)	2(5)	3(7)	6(15)
	M (#)	3(5)	4(7)	9(15)
	F (%)	40	43	40
	M (%)	60	57	60

SUPPLEMENTAL TABLE 6. Patient information for swab and BALF samples used for RNA-Seq. Related to FIGURE and SUPPLEMENTAL FIGURE 5. Age and sex characteristics of patient cohorts analyzed in **Figure 5 and Supplemental Figure 5**. Nasopharyngeal swabs and BALF from SARS-CoV-2 positive patients that were either home-isolated (HI), hospitalized (HOSP)) or hospitalized and admitted to the ICU (ICU) and BALF from SARS-CoV-2 negative patients were analyzed by RNA-Seq. Q1=quartile 1, Q3=quartile 3, IQR=interquartile range, , F=female, M=male, #=number of samples, %=percentage of samples.

		COVID-19	ARDS H1N1+	ARDS H1N1-	Fibrosis	Sarcoidosis	Transplant
	**Samples (#)**	29(68)	5(68)	4(68)	10(68)	10(68)	10(68)
**Age**	**Minimum**	28	42	44	64	35	35
	**Maximum**	79	67	76	83	77	67
	**Q1**	56	54	54	66	39	53
	**Q3**	66	62	70	74	53	58
	**IQR**	10	8	16	8	14	5
	**Median**	60	59	63	70	46	56
	**Mean**	61	57	61	71	50	53
**Sample**	**BALF (#)**	29(29)	5(5)	4(4)	10(10)	10(10)	10(10)
	**Plasma (#)**	17(29)	0(5)	0(4)	0(10)	0(10)	0(10)

SUPPLEMENTAL TABLE 7. Patient information for BALF and plasma samples used for cytokine quantification. Related to FIGURE and SUPPLEMENTAL FIGURE 6. Age and type of collected sample of patient cohorts analyzed in **Figure and Supplemental Figure 6**. BALF and plasma from patients with COVID-19 was analyzed. BALF from patients suffering from: non-COVID-19 ARDS (divided in H1N1 Influenza A virus positive or not), fibrosis, sarcoidosis, and that received lung transplant was analyzed. Q1=quartile 1, Q3=quartile 3, IQR=interquartile range.
